# Carnitine palmitoyltransferase 1A promotes mitochondrial fission by enhancing MFF succinylation in ovarian cancer

**DOI:** 10.1038/s42003-023-04993-x

**Published:** 2023-06-08

**Authors:** Yaqin Zhu, Yue Wang, Ying Li, Zhongqi Li, Wenhui Kong, Xiaoxuan Zhao, Shuting Chen, Liting Yan, Lenan Wang, Yunli Tong, Huanjie Shao

**Affiliations:** National Engineering Laboratory for Resource Developing of Endangered Chinese Crude Drugs in Northwest of China, Key Laboratory of the Ministry of Education for Medicinal Resources and Natural Pharmaceutical Chemistry, College of Life Sciences, Shaanxi Normal University, 710119 Xi’an, Shaanxi China

**Keywords:** Oncogenes, Mitochondria

## Abstract

Mitochondria are dynamic organelles that are important for cell growth and proliferation. Dysregulated mitochondrial dynamics are highly associated with the initiation and progression of various cancers, including ovarian cancer. However, the regulatory mechanism underlying mitochondrial dynamics is still not fully understood. Previously, our study showed that carnitine palmitoyltransferase 1A (CPT1A) is highly expressed in ovarian cancer cells and promotes the development of ovarian cancer. Here, we find that CPT1A regulates mitochondrial dynamics and promotes mitochondrial fission in ovarian cancer cells. Our study futher shows that CPT1A regulates mitochondrial fission and function through mitochondrial fission factor (MFF) to promote the growth and proliferation of ovarian cancer cells. Mechanistically, we show that CPT1A promotes succinylation of MFF at lysine 302 (K302), which protects against Parkin-mediated ubiquitin-proteasomal degradation of MFF. Finally, the study shows that MFF is highly expressed in ovarian cancer cells and that high MFF expression is associated with poor prognosis in patients with ovarian cancer. MFF inhibition significantly inhibits the progression of ovarian cancer in vivo. Overall, CPT1A regulates mitochondrial dynamics through MFF succinylation to promote the development of ovarian cancer. Moreover, our findings suggest that MFF is a potential therapeutic target for ovarian cancer.

## Abstract (web summary)

Carnitine palmitoyltransferase 1A is found to promote the growth and proliferation of ovarian cancer cells through regulating mitochondrial dynamics by succinylating mitochondrial fission factor at lysine 302.

## Introduction

Mitochondria are highly dynamic organelles that continuously fuse and divide in the cell. The dynamics of mitochondrial fusion and fission are strictly regulated by some large GTPase proteins, such as optic atrophy 1 (OPA1)^1^, mitofusin 1 (MFN1) and mitofusin 2 (MFN2), which are involved in regulating mitochondrial inner and outer membrane fusion, and by dynamin-related protein 1 (DRP1) and its receptor proteins mitochondrial fission factor (MFF), Fis1, and MiD49/51, which are involved in regulating mitochondrial fission^2–4^. Once the dynamic balance of mitochondrial fusion and fission has become abnormal, cells undergo pathological changes, such as tumorigenesis. Studies have shown that mitochondria in normal cells are mostly linear and reticular, while mitochondria in tumor cells are mostly fissioned and granular^5^. Our previous work also showed that enhanced mitochondrial fission or weakened fusion in non-small-cell lung cancer (NSCLC) played an important role in promoting the onset and development of NSCLC^6^. Abnormal mitochondrial dynamics play an important role in the initiation, development and drug resistance of ovarian cancer. It has been reported that mitochondrial biogenesis is significantly enhanced in ovarian cancer cells compared to normal cells^7^. Mitochondrial morphology changes from a filamentous network to a single enlarged organelle with increasing malignancy in serous ovarian cancer^8^. In addition, in doxorubicin-resistant ovarian cancer cells, mitochondrial morphology and subcellular localization have been observed to be significantly changed: the mitochondria became rounder and more uniformly distributed in cells^9^.

Abnormal mitochondrial dynamics are usually associated with aberrant expression or activation of mitochondrial fusion or fission-related proteins. For example, MFF, a key receptor for recruiting Drp1 from the cytosol to the mitochondrion, is highly expressed in breast cancer, liver cancer and colon cancer and promotes cancer progression^10–12^. The high expression of MFF also contributes to cancer drug resistance^13^. In addition, MFF can be phosphorylated by kinases such as AMP-activated protein kinase (AMPK) at sites of Ser155 and Ser172, which facilitate the recruitment of DRP1 to mitochondria and enhance mitochondrial fission in response to energy stress^14^. Although the role of abnormal mitochondrial dynamics in cancer development and multidrug resistance has been confirmed in many ways, the regulatory mechanism of mitochondrial dynamics is still not fully understood.

Uptake of fatty acids (FAs) has been reported to allow cancer cells to survive energy stress conditions by upregulating fatty acid oxidation (FAO)^15–17^. FAs can trigger mitochondrial fragmentation to alter cellular metabolic pathways in colon cancer cells^12^. As a key rate-limiting enzyme for the intracellular transport of FAs to the mitochondrial matrix for FAO, carnitine palmitoyltransferase 1A (CPT1A) is highly expressed in various malignant tumors and promotes tumor progression. CPT1A knockdown was reported to alter mitochondrial morphology and suppress cell proliferation in BT549 cells^18^. Whether CPT1A can promote cancer progression by directly regulating mitochondrial dynamics is still unclear. As reported, CPT1A not only promotes the adaptation of cancer cells to the abnormal tumor microenvironment through the production of ATP and NADPH by FAO^19,20^ but also participates in the regulation of the expression of key pathways and factors that regulate cell gene expression and apoptosis^21,22^. Recent studies have found that in addition to carnitine palmitoyltransferase (CPTase) activity, CPT1A itself also has lysine succinyltransferase (LSTase) activity^23^ and can regulate the function and stability of substrate proteins by promoting the succinylation modification of substrate proteins^24–26^.

Our previous study showed that CPT1A is highly expressed in ovarian cancer tissue cells and promotes the occurrence and development of ovarian cancer^27^. In this study, we found that CPT1A can promote the succinylation of MFF through its lysine succinyltransferase activity, regulate mitochondrial dynamics and promote the growth and proliferation of ovarian cancer cells. Our findings reveal new role and molecular mechanisms by which CPT1A regulates mitochondrial dynamics to promote ovarian cancer progression through modulation of MFF succinylation.

## Results

### CPT1A regulates mitochondrial dynamics through MFF

Consistent with previous studies^27^, knocking down CPT1A significantly inhibited the growth of ovarian cancer cells in vitro and in vivo (Supplementary Fig. 1a–d). To test whether this inhibition is solely dependent on CPT1A-mediated transport of long-chain fatty acids, we overexpressed CPT1A-G710E, a carnitine palmitoyltransferase (CPTase)-deficient mutant^23,28^, in SKOV-3 cells with a CPT1A knockdown. As shown in Supplementary Fig. 1e–f, the expression of CPT1A-G710E did not restore cellular ATP but significantly rescued the growth and proliferation of ovarian cancer cells, suggesting that the decrease in cellular ATP by CPT1A knockdown may not be the only mechanism for the inhibition of cell growth and proliferation.

**Fig. 1:**
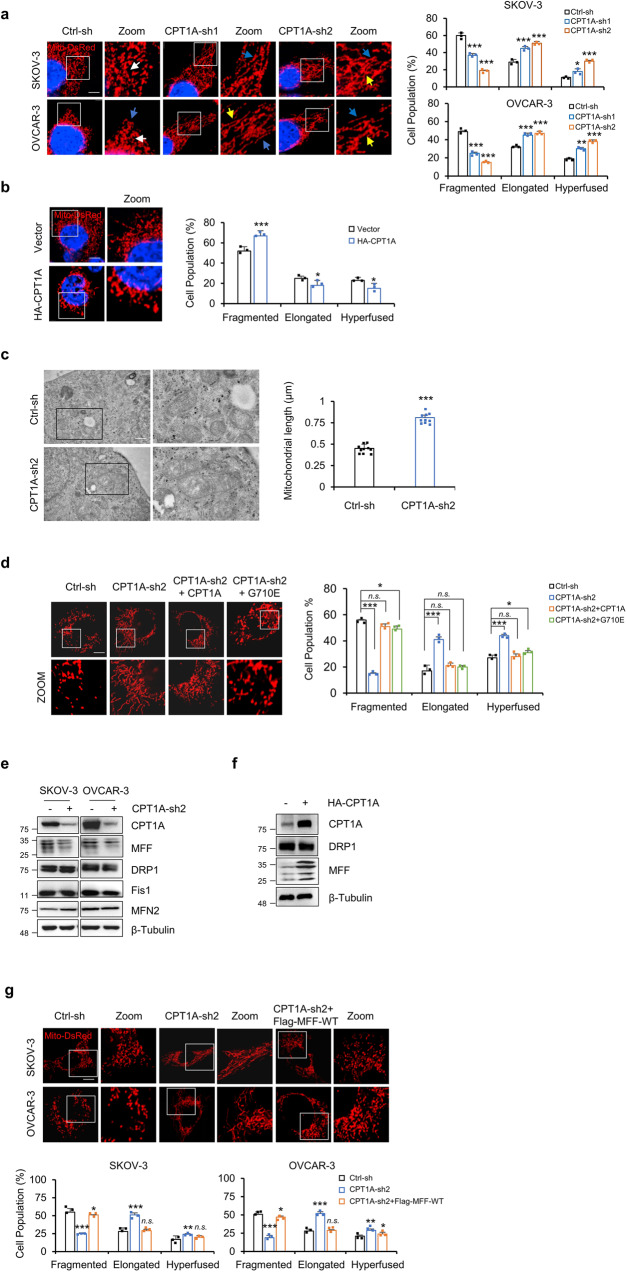
CPT1A regulates mitochondrial dynamics through MFF. **a** Representative confocal microscopy images of the mitochondria in SKOV-3 and OVCAR-3 cells with control or CPT1A knockdown (left, scale bar = 10 μm). The proportion of cells with different mitochondrial morphologies was quantified (*n* = 3 samples, each sample contains 100 cells, right). The white arrow represents fragmented mitochondria, the blue arrow represents elongated mitochondria, and the yellow arrow represents hyperfused mitochondria. **b** Representative images of the mitochondrial network in SKOV-3 cells with empty vector or exogenous CPT1A overexpression (HA-CPT1A) (left, scale bar = 10 μm). The proportion of cells (*n* = 3 samples, each sample contains 100 cells) with fragmented, elongated, and hyperfused mitochondria was quantified (right). **c** Representative images showing mitochondrial morphology in SKOV-3 cell with ctrl-sh, CPT1A knockdown by transmission electron microscopy (*n* = 10 for each group). Scale bars = 500 nm. **d** CPT1A-WT and CPT1A-G710E overexpression rescued CPT1A downregulation-induced mitochondrial fusion and restored mitochondrial fragmentation in SKOV-3 cells, *n* = 3 samples, each sample contains 100 cells. Scale bar = 10 μm. Data represent the mean ± SD. *p < 0.05, **p < 0.01 and *n.s*. indicates no significant difference compared with the control groups. **e** CPT1A was knocked down by CPT1A-sh2 in SKOV-3 or OVCAR-3 cells. Mitochondrial fission- and fusion-related proteins were examined by western blotting. Bands are derived from the different gels. **f** CPT1A was exogenously overexpressed in SKOV-3 cells. CPT1A, DRP1 and MFF were examined. β-Tubulin was included as a protein loading control. **g** Representative confocal microscopy images of the mitochondrial network in SKOV-3 and OVCAR-3 cells exogenously overexpressed MFF in the background of CPT1A knockdown (upper, scale bar = 10 μm). The proportion of cells with different mitochondrial morphologies was quantified (*n* = 3 samples, each sample contains 100 cells, lower). All data represent the mean ± SD; *p < 0.05, **p < 0.01, ***p < 0.001; *n.s*. indicates no significant difference compared with the control groups. Scale bar = 10 μm.

Mitochondria are highly dynamic organelles that are important for cell growth and proliferation. To explore whether CPT1A is involved in the regulation of mitochondrial dynamics, we knocked down CPT1A in SKOV-3 and OVCAR-3 cells. As shown in Fig. 1a, CPT1A silencing significantly promoted mitochondrial fusion and led to the elongation and hyperfusion of mitochondria. For instance, in SKOV-3 cells, CPT1A-sh2 resulted in a decrease in mitochondrial fragmentation from 60% to 19% in comparison to Ctrl-sh, while elongated and hyperfused mitochondria increased from 29% to 56% and from 11% to 25%, respectively. In addition, knockdown of CPT1A significantly promoted mitochondrial fusion in xenograft tumors in vivo (Supplementary Fig. 1g). On the other hand, exogenously overexpressed HA-tagged CPT1A in SKOV-3 cells caused mitochondria to appear more fragmented (Fig. 1b and Supplementary Fig. 1h). Furthermore, the effects of CPT1A on mitochondrial morphology were further confirmed with transmission electron microscopy (TEM). Mitochondria showed a significant increase in length, from an average of 0.45 to 0.81 μm after CPT1A knockdown and a significant decrease in length from an average of 0.48 to 0.34 μm after CPT1A exogenous overexpression (Fig. 1c and Supplementary Fig. 1i). Finally, exogenous overexpression of CPT1A-WT or CPT1A-G710E restored the fission of mitochondria caused by CPT1A knockdown (Fig. 1d and Supplementary Fig. 1j) further suggesting that CPT1A regulates mitochondrial dynamics.

To explore the mechanism by which CPT1A regulates the dynamics of mitochondria, we analyzed the key players involved in mitochondrial fission and fusion. Among all of the proteins examined, MFF was dramatically decreased after CPT1A knockdown in SKOV-3 and OVCAR-3 cells (Fig. 1e). In addition, exogenous overexpression of CPT1A in SKOV-3 cells significantly upregulated MFF (Fig. 1f). To verify whether MFF is the key mediator of CPT1A in regulating mitochondrial fusion and fission dynamics, we exogenously overexpressed MFF in the background of CPT1A knockdown (Supplementary Fig. 1k). MFF overexpression significantly restored mitochondrial fission (Fig. 1g). Interestingly, overexpressing CPT1A-G710E, the CPTase-deficient mutant, also restored the expression of MFF (Supplementary Fig. 1l) and rescued mitochondrial morphology (Fig. 1d).

As reported, MFF is a key player to recruit DRP1 for mitochondrial fission^29^. Interestingly, we did not observe significant change in DRP1 expression in SKOV-3 or OVCAR-3 cells with CPT1A knockdown or exogenous overexpression (Fig. 1e, f). To investigate whether CPT1A effects DRP1 recruitment to mitochondria, we analyzed DRP1 subcellular localization in the background of CPT1A knockdown or overexpression. The results showed that CPT1A knockdown reduced DRP1 recruitment to mitochondria, while CPT1A overexpression significantly enhanced the colocalization of DRP1 with mitochondria (Supplementary Fig. 1m). Taken together, our results indicate that CPT1A does not affect DRP1 protein expression, but significantly affects DRP1 recruitment to mitochondria.

As reported, MFF phosphorylation activation by AMPK is important for DRP1 recruitment to mitochondria and thus mitochondria fission^14^. As an essential cellular energy sensor, AMPK is activated by the reduction of intracellular ATP concentration^30^. CPT1A knockdown resulted in a decrease in cellular ATP (Supplementary Fig. 1e). Therefore, we examined the activation of AMPK and the expression of p-MFF (Ser172), and found that AMPK was activated upon CPT1A knockdown (Supplementary Fig. 1l). Similar to the changes in total MFF protein, p-MFF was decreased in the CPT1A knockdown group and significantly increased in the CPT1A-sh + G710E group compared with the control group (Supplementary Fig. 1l). Interestingly, after normalizing the expression of p-MFF with total MFF, the results showed that the ratio of p-MFF/MFF was significantly upregulated in both the CPT1A-sh and CPT1A-sh + G710E groups, suggesting that AMPK activation promoted MFF phosphorylation. Together with the results that CPT1A affects DRP1 recruitment to mitochondria, we hypothesize that Drp1 recruitment to mitochondria is closely related to the presence of total MFF in ovarian cancer cells and that CPT1A regulates mitochondrial dynamics by regulating MFF expression.

### CPT1A promotes the growth and proliferation of ovarian cancer cells by regulating mitochondrial dynamics

**Fig. 2:**
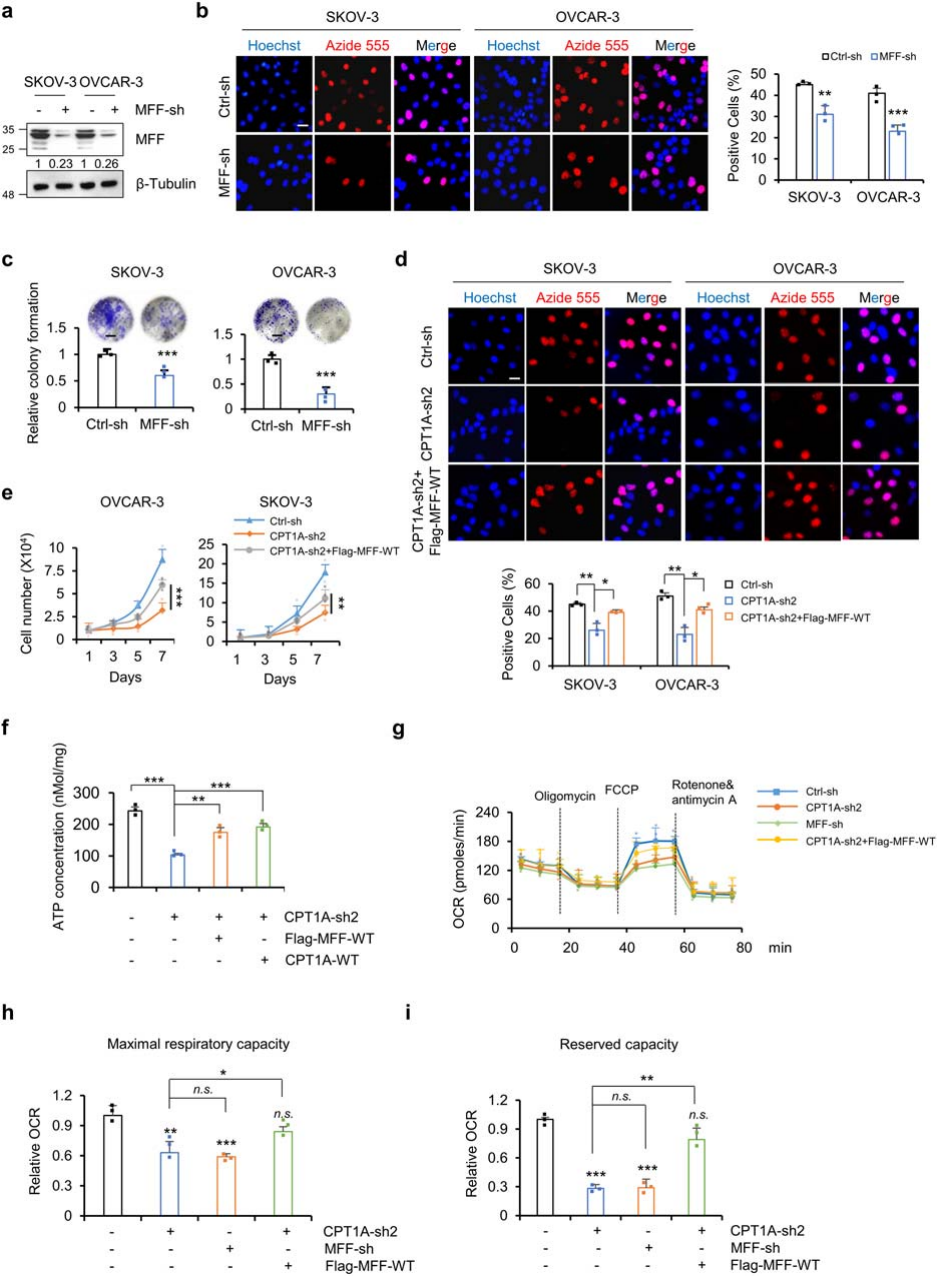
CPT1A promotes the growth and proliferation of ovarian cancer cells by regulating mitochondrial dynamics. **a** Western blot analysis of MFF and β-tubulin in SKOV-3 and OVCAR-3 cells. Relative MFF expression was normalized to β-tubulin and then compared to control groups. **b** The EdU proliferation assay was performed in SKOV-3 and OVCAR-3 cells with control or MFF knockdown. Representative images (left) and the ratio of EdU-positive SKOV-3 and OVCAR-3 cells (right) are shown, *n* = 3 for each group. **c** Colony formation assay was performed in SKOV-3 and OVCAR-3 cells with control and MFF knockdown. Microphotographs covering representative areas of each treatment are shown (upper). The number of colonies in each case was analyzed (*n* = 3 for each group, lower). Scale bars, 2 mm. **d** Cell proliferation was determined by EdU assay in SKOV-3 and OVCAR-3 cells with control, CPT1A knockdown and exogenous overexpression of MFF in conjunction with CPT1A knockdown. Representative images (upper) and the ratio of EdU-positive (azide-555, red) SKOV-3 cells (lower) are shown, *n* = 3 for each group. **e** Cells described in (**d**) were seeded in 24-well plates and harvested for counting by the trypan blue assay for up to 7 days, *n* = 3 for each group. **f** ATP content detection in SKOV-3 cells with control, CPT1A knockdown, or MFF or CPT1A-WT overexpression in conjunction with CPT1A knockdown, *n* = 3 for each group. **g** The oxygen consumption rate (OCR) assay was performed in SKOV-3 cells with control, CPT1A knockdown, MFF knockdown or exogenous overexpression of MFF in conjunction with CPT1A knockdown. The relative OCR of maximal respiratory capacity (**h**) and reserved capacity (**i**) were quantified, *n* = 3 for each group. All data represent the mean ± SD; *p < 0.05, **p < 0.01, ***p < 0.001; *n.s*. indicates no significant difference compared with the control groups. Scale bars, 25 μm.

As has been reported, mitochondrial dynamics occur in a regulated manner to maintain cellular energy and metabolic homeostasis and play an important role in cell growth and proliferation. As shown in Fig. 2a–c and Supplementary Fig. 2a, MFF knockdown significantly inhibited cell proliferation and clone formation in SKOV-3 and OVCAR-3 cells. To investigate whether CPT1A promotes cell growth and proliferation by regulating mitochondrial dynamics, we overexpressed MFF in SKOV-3 and OVCAR-3 cells with CPT1A knockdown. The results showed that MFF overexpression rescued cell growth and proliferation (Fig. 2d, e). MFN2 is a mitochondrial fusion-related factor. Interestingly, we knocked down MFN2 in the background of CPT1A knockdown in SKOV-3 cells and found that mitochondrial fission was restored and cell growth was partially rescued (Supplementary Fig. 2b–d). We previously found that CPT1A inactivation could induces G0/G1 cell cycle arrest and upregulation of p21^27^. Here, similar to CPT1A knockdown, MFF inactivation also resulted in a significant upregulation of p21 expression (Supplementary Fig. 2e, f). This suggests that the mitochondrial fusion and fission morphology is closely related to ovarian cancer cell growth and proliferation, and that CPT1A might promote cell growth and proliferation by regulating mitochondrial dynamics.

The morphology of mitochondria is closely related to ATP production and ROS generation^31^. Similar to the knockdown of CPT1A, MFF knockdown significantly led to a decrease in cellular ATP (Supplementary Fig. 2g). In addition, MFF and CPT1A-WT overexpression in SKOV-3 cells significantly restored the cellular ATP reduction caused by CPT1A knockdown (Fig. 2f). Furthermore, by utilizing the Seahorse Mito Stress Assay, we measured the overall oxygen consumption rate (OCR) and found that knockdown of CPT1A significantly reduced the maximum respiratory capacity and reserved capacity in SKOV-3 cells, which could be restored by exogenous overexpression of MFF (Fig. 2g–i), suggesting that CPT1A knockdown regulates mitochondrial dynamics and, thus, mitochondrial function through MFF. Furthermore, we overexpressed CPT1A in SKOV-3 cells with MFF knockdown. The results showed that overexpression of CPT1A in conjunction with MFF-sh did not rescue mitochondrial morphology (Supplementary Fig. 2h, i), cell proliferation (Supplementary Fig. 2j, k) or ATP (Supplementary Fig. 2l). OCR analysis showed that overexpression of CPT1A in conjunction with MFF-sh only slightly restored the maximal respiratory capacity and reserved the respiratory capacity of mitochondria (Supplementary Fig. 2m–o). Together, these results further demonstrate that CPT1A regulates mitochondrial dynamics by regulating MFF expression, which in turn promotes mitochondrial function and cell growth in ovarian cancer cells.

### CPT1A stabilizes MFF by inhibiting its ubiquitination

**Fig. 3:**
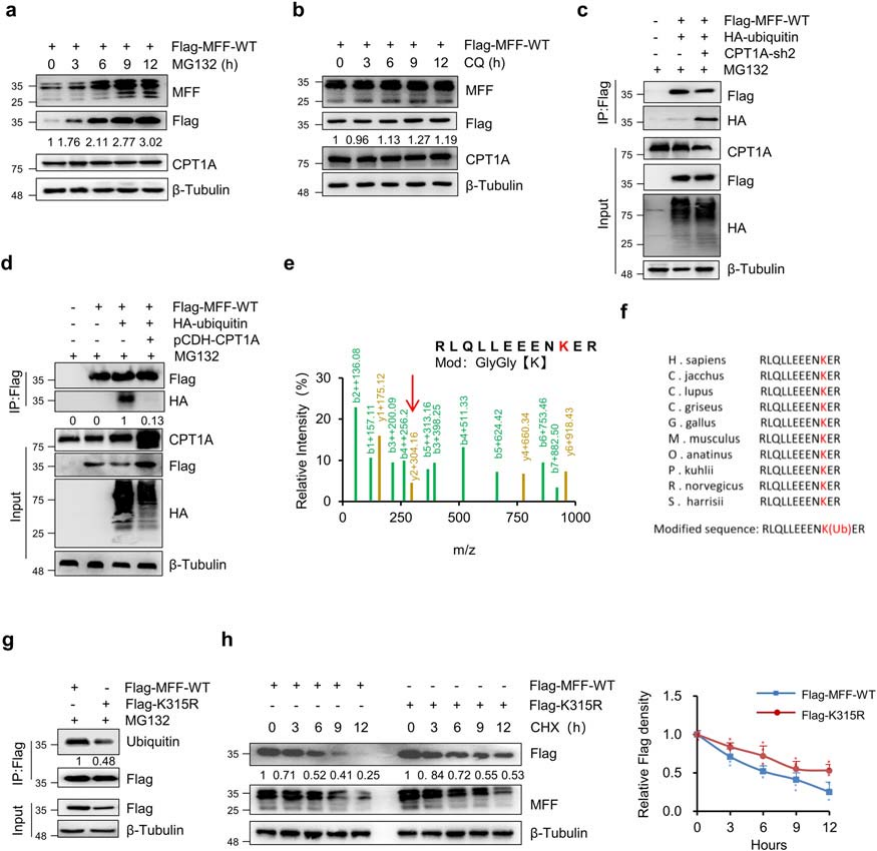
CPT1A inhibits the ubiquitination of MFF to stabilize MFF. **a**, **b** SKOV3 cells exogenously expressing Flag-MFF-WT were treated with 10 μM MG132 (**a**) or 5 μM chloroquine (CQ, **b**) for up to 12 h, and the expression of endogenous MFF, Flag-MFF and CPT1A was assessed by immunoblotting. Relative expression of Flag was normalized to β-tubulin and then compared to 0 h. **c** SKOV-3 cells cotransfected with plasmids as indicated were treated with MG132 (10 μM) for 8 h. Cells were harvested for immunoprecipitation with anti-Flag antibody. Bands are derived from the different gels. **d** 293TN cells cotransfected with plasmids as indicated were treated with MG132 (10 μM) for 8 h. Cells were harvested for immunoprecipitation with anti-Flag antibody. The relative HA expression was normalized to Flag (IP) and then compared to control groups. **e** Mass spectrometry analysis of MFF ubiquitinated peptides. The red arrow points to the parental ions of interest subjected to tandem MS analysis, where the ubiquitination site occurs. **f** Multi-species conservation analysis of ubiquitination modified sequences. **g** 293TN cells transfected with Flag-MFF-WT or Flag-MFF-K315R (Flag-K315R) plasmids were treated with MG132 (10 μM) for 8 h. Cells were harvested for immunoprecipitation with anti-Flag antibody and immunoblotting with anti-Flag and anti-ubiquitin antibodies. The relative ubiquitination of MFF was determined by normalizing ubiquitin to Flag (IP). **h** 293TN cells transfected with Flag-MFF-WT or Flag-MFF-K315R (Flag-K315R) were treated with 10 μM cycloheximide (CHX) for up to 12 h. Cells were harvested for immunoblotting with anti-Flag and anti-MFF antibodies. Beta-Tubulin was used as a loading control. Bands are derived from the different gels. Densitometric analysis of the Flag immunoblot band was performed with ImageJ (*n* = 3 for each group). Data represent the mean ± SD; *p < 0.05, **p < 0.01, ***p < 0.001, *n.s*. indicates no significant difference compared with the control groups.

To explore how CPT1A knockdown leads to MFF degradation, we treated SKOV-3 and OVCAR-3 cells with cycloheximide (CHX) for up to 9 h in the presence or absence of CPT1A. The results showed that CPT1A knockdown significantly enhanced the degradation of MFF (Supplementary Fig. 3a). In eukaryotic cells, protein degradation is mainly mediated through the ubiquitin-proteasome pathway or lysosomal proteolysis. To further confirm how CPT1A regulates the stability of MFF, we treated SKOV-3 cells with exogenous expression of Flag-MFF with the proteasome inhibitor MG132 or the lysosomal pathway inhibitor chloroquine separately. MG132 treatment significantly led to the accumulation of Flag-MFF (Fig. 3a), while chloroquine did not (Fig. 3b). The results were also observed in ES2 cells (Supplementary Fig. 3b and Supplementary Fig. 3c), suggesting that the degradation of MFF through CPT1A knockdown may be related to ubiquitin-proteasome degradation.

Then, Flag-MFF and HA-ubiquitin were exogenously overexpressed in SKOV-3 cells to verify whether CPT1A regulates the ubiquitination of MFF. The results showed that CPT1A knockdown significantly increased the ubiquitination level of MFF (Fig. 3c), while the exogenous overexpression of CPT1A significantly inhibited the ubiquitination of MFF (Fig. 3d), suggesting that the presence of CPT1A protein inhibits the ubiquitination of MFF and affects the stability of MFF.

To determine the ubiquitination site of MFF, we enriched MFF by immunoprecipitation (IP) for mass spectrometry analysis. As shown in Fig. 3e, the MFF K315 site was modified by ubiquitination, suggesting that K315 may be an ubiquitination site regulated by CPT1A. The conservation of the K315 site was analyzed, and it was found that this site is highly conserved in MFF across various species (Fig. 3f). To examine whether the ubiquitination of K315 affects the stability of MFF, we mutated K315 to arginine (R). The K315R mutation significantly decreased the ubiquitination of MFF and suppressed MFF degradation (Fig. 3g, h). On the other hand, overexpression of K315R in conjunction with CPT1A knockdown rescued the growth and proliferation of SKOV-3 cells (Supplementary Fig. 3d, e) and promoted mitochondrial fission (Supplementary Fig. 3f). Together, these results suggest that CPT1A inhibits the ubiquitination of MFF K315, thereby preventing MFF degradation.

### Parkin promotes the ubiquitination of MFF

**Fig. 4:**
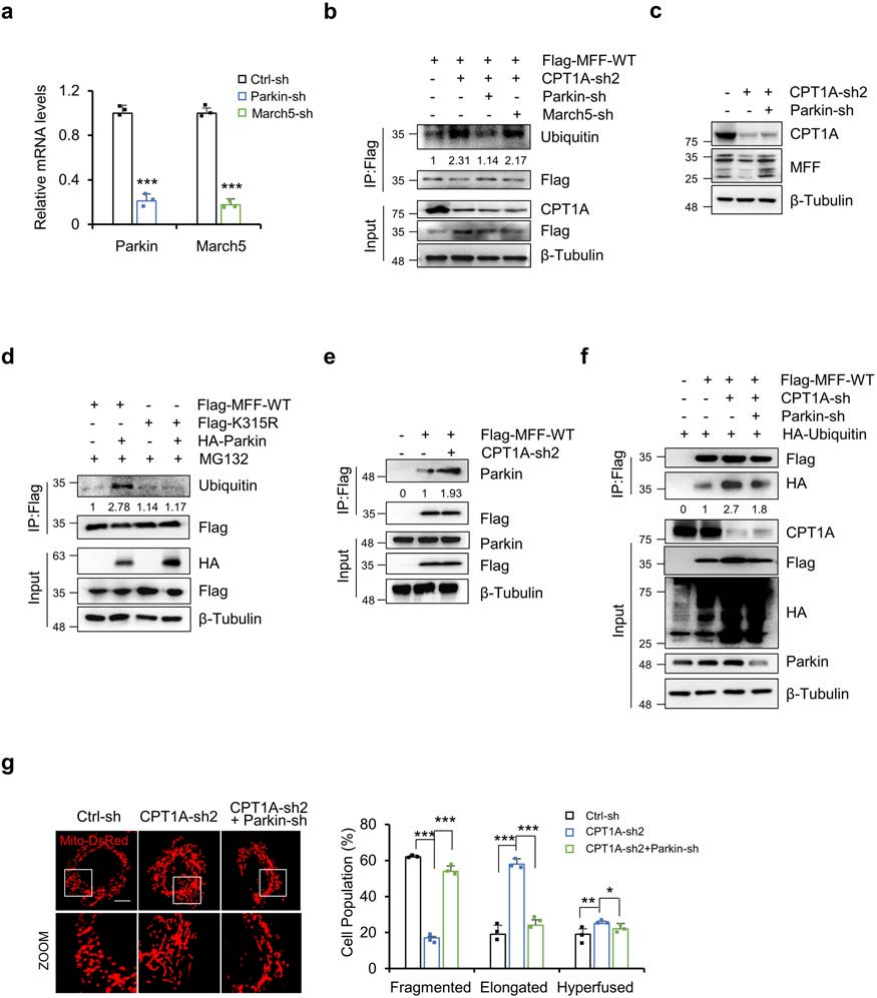
Parkin promotes the ubiquitination of MFF. **a** The shRNA knockdown efficiency of Parkin and March5 was examined in SKOV-3 cells by real time PCR (*n* = 3 for each group). **b** SKOV-3 cells exogenously expressing Flag-MFF were knocked down with CPT1A, March5 or Parkin as indicated. Cells were harvested for immunoprecipitation with an anti-Flag antibody, followed by immunoblotting with an anti-Flag or anti-ubiquitin antibody, and the relative ubiquitin expression was normalized to Flag (IP) and then compared to control groups. **c** MFF was examined by WB in SKOV-3 cells with control, CPT1A knockdown or CPT1A and Parkin double knockdown. **d** SKOV-3 cells co-transfected with plasmids as indicated were treated with MG132 (10 μM) for 8 h. Cells were harvested for immunoprecipitation with an anti-Flag antibody. **e** SKOV-3 cells exogenously expressing Flag-MFF-WT were knocked down with CPT1A as indicated. Cells were harvested for immunoprecipitation with anti-Flag antibody, followed by immunoblotting with anti-Flag or anti-Parkin antibody. The relative Parkin expression was normalized to Flag (IP) and then compared to control groups. **f** SKOV-3 cells cotransfected with plasmids as indicated. Cells were harvested for immunoprecipitation with an anti-Flag antibody. Then, immunoblotting was performed with anti-Flag or anti-HA antibodies. The relative HA expression was normalized to Flag (IP) and then compared to control groups. **g** Representative confocal microscopy images of mitochondria in SKOV-3 cells with control, CPT1A knockdown or CPT1A and Parkin double knockdown (left). The proportion of cells with different mitochondrial morphologies was quantified (*n* = 3 samples, each sample contains 100 cellsright). Data represent the mean ± SD; *p < 0.05, **p < 0.01, ***p < 0.001, *n.s*. indicates no significant difference compared with the control groups. Scale bars, 10 μm.

As reported, E3 ubiquitin ligases, including Parkin^32,33^ and March5^34^, promote the ubiquitination of MFF. We found that Parkin knockdown significantly inhibited the ubiquitination of MFF, while knockdown of March5 had little effect on the ubiquitination of MFF (Fig. 4a, b), indicating that Parkin may mediate MFF ubiquitination in ovarian cancer cells. Furthermore, Parkin knockdown rescued MFF expression in SKOV-3 cells in the background of CPT1A knockdown (Fig. 4c). The MFF K315R mutation significantly reduced the ubiquitination modification of MFF (Fig. 4d), suggesting that Parkin may promote the ubiquitination of MFF at K315. To investigate how CPT1A affects the ubiquitination of MFF, we evaluated the interaction between Parkin and MFF by IP and found that CPT1A knockdown significantly enhanced the interaction between Parkin and MFF (Fig. 4e), suggesting that CPT1A might regulate the ubiquitination of MFF by regulating the interaction between Parkin and MFF. To further confirm the role of Parkin in MFF ubiquitination by CPT1A knockdown, we examined the ubiquitination of MFF in Parkin knockdown in conjunction with CPT1A-sh. The results showed that Parkin knockdown dramatically reduced the ubiquitination of Flag (MFF) induced by CPT1A knockdown (Fig. 4f), demonstrating that Parkin is involved in CPT1A regulation of MFF ubiquitination.

Our results show that CPT1A knockdown leads to the degradation of MFF and promotes mitochondrial fusion. Next, we silenced Parkin by shRNA in the background of CPT1A knockdown in SKOV-3 cells. The results showed that Parkin knockdown restored the expression of MFF and decreased mitochondrial fusion caused by CPT1A knockdown (Fig. 4c, g). Furthermore, the growth and proliferation of ovarian cancer cells were also significantly restored with Parkin knockdown (Supplementary Fig. 4). Altogether, these results suggest that CPT1A promotes ovarian cancer cell proliferation by inhibiting Parkin-mediated ubiquitin-proteasome degradation of MFF.

### CPT1A promotes MFF succinylation and inhibits its ubiquitin-proteasome degradation

**Fig. 5:**
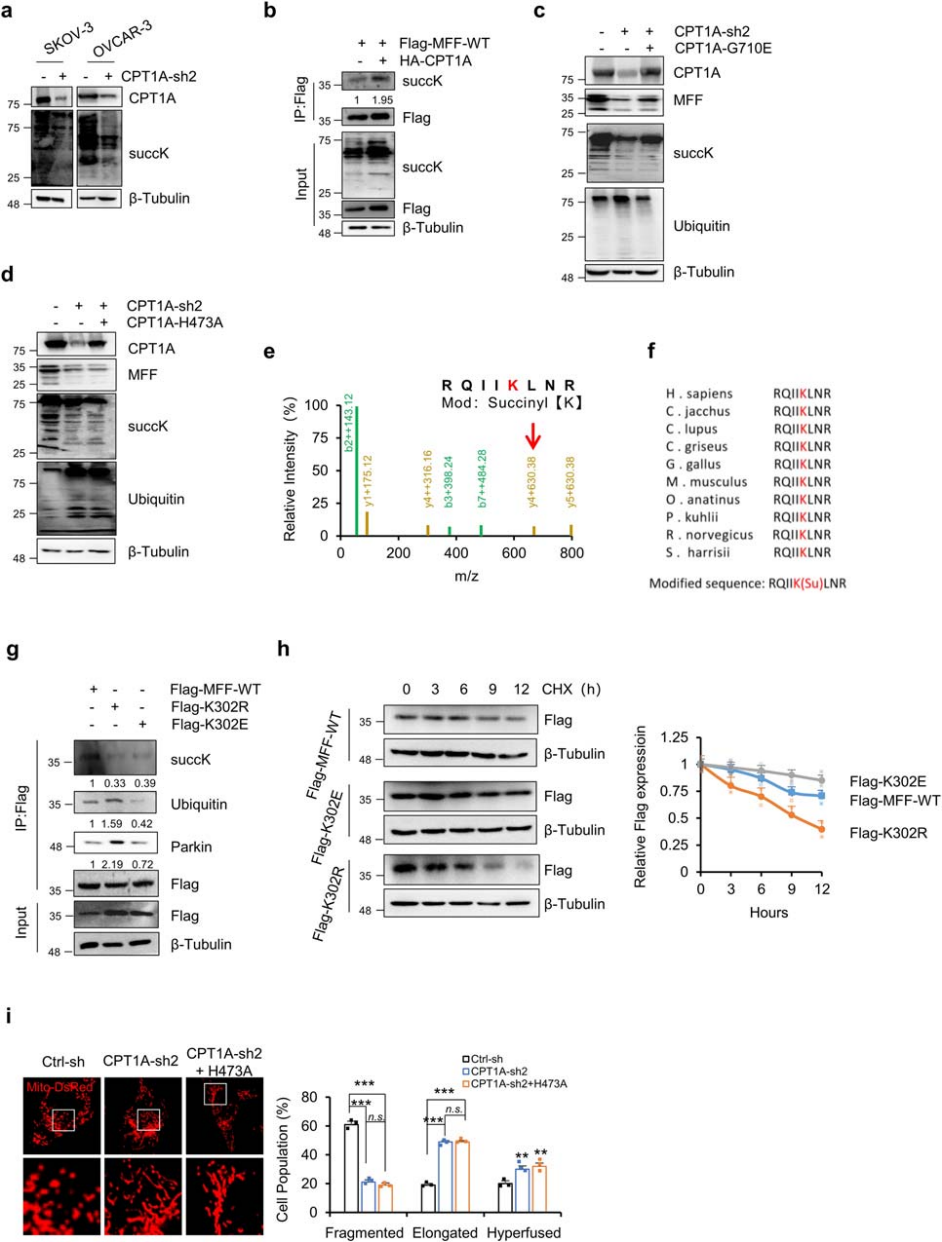
CPT1A promotes MFF succinylation and inhibits its ubiquitin-proteasome degradation. **a** Succinylated lysine (succK) was examined in SKOV-3 and OVCAR-3 cells with or without CPT1A knockdown. Bands are derived from the different gels. **b** ES2 cells exogenously expressing Flag-MFF were infected with the HA-CPT1A lentivirus as indicated. Cells were harvested for immunoprecipitation with an anti-Flag antibody. Succinylated lysine (succK) was examined by Western blotting. The relative succK expression was normalized to Flag (IP) and then compared to control groups. **c**, **d** The CPT1A-G710E (G710E, **c**) or CPT1A-H473A (H473A, **d**) was transfected into CPT1A-knockdown SKOV-3 cells. CPT1A, MFF, and succinylation and ubiquitination of cellular proteins were analyzed by Western blotting. Beta-tubulin was used as an endogenous control. **e** Mass spectrum analysis of MFF succinylated peptides. Red arrows point to the parent ion of interest analyzed by tandem mass spectrometry where succinylation occurs. **f** Conservation analysis of amino acids in the region near the succinylation site among species. **g** SKOV-3 cells transfected with Flag tagged wild type MFF (Flag-WT), MFF K302R (Flag-K302R) or MFF K302E (Flag-K302E) were immunoprecipitated with an anti-Flag antibody, followed by immunoblotting with anti-Flag, anti-succK, anti-Parkin or anti-ubiquitin antibodes. The relative succK, ubiquitin and parkin expressions were normalized to Flag (IP) and then compared to control groups. **h** 293TN cells transfected with Flag-MFF-WT, Flag-K302R or Flag-K302E were treated with CHX (10 μM) for up to 12 h. Whole cell lysates were prepared and assessed using anti-Flag antibodies (left). Relative expression of Flag-MFF was normalized to β-tubulin and then compared to 0 h (*n* = 3 for each group, right). Bands are derived from the different gels. **i** Representative confocal microscopy images of the mitochondria in SKOV-3 cells with control, CPT1A-knockdown, or exogenous overexpression of CPT1A H473A in conjunction with CPT1A-knockdown (left). The proportion of cells with different mitochondrial morphologies was quantified (*n* = 3 samples, each sample contains 100 cells, right). Data represent the mean ± SD; ***p < 0.001, *n.s*. indicates no significant difference compared with the control groups or as indicated. Scale bars, 10 μm.

Protein ubiquitination can also be regulated by other types of protein posttranslational modifications, such as phosphorylation, acetylation, propionylation, butylation, glutarylation and succinylation^35^. To explore how CPT1A regulates the ubiquitination modification of MFF by Parkin, we analyzed the acylation of cell protein after knockdown or inhibition of CPT1A and observed that CPT1A knockdown significantly reduced the succinylation of the total cell protein but had little effect on other acylation modifications, such as acetylation, propionylation, butylation, and glutarylation (Fig. 5a, Supplementary Fig. 5a). As reported recently, CPT1A can promote the succinylation of lysine residues of its substrate^23^. The exogenous expression of CPT1A and Flag-tagged MFF in ES2 cells showed that CPT1A expression significantly increased the succinylation of MFF compared with that of the vector control (Fig. 5b). In addition, the interaction between CPT1A and MFF was validated by coimmunoprecipitation in SKOV-3 cells (Supplementary Fig. 5b).

As reported, CPTase activity and LSTase activity are independently regulated by CPT1A^23^. CPT1A-H473A, a catalytically inactive mutant that disrupts the putative binding pocket for the sulfur atom of the acyl-CoA thioester and lacks both CPTase activity and LSTase activity, and CPT1A-G710E, a CPTase-deficient mutant, were transfected into CPT1A-knockdown cells separately. The results showed that CPT1A-G710E restored succinylation and inhibited ubiquitination and degradation of MFF (Fig. 5c). In contrast, CPT1A-H473A neither restored succinylation of cellular proteins nor restored MFF protein expression (Fig. 5d).

To determine the specific succinylated lysine (succK) in MFF, MFF was enriched by IP for In-gel protein digestion and LC-MS/MS analysis. We found that MFF protein was succinylated at lysine 302 (K302) in SKOV-3 cells (Fig. 5e). Similar to that reported by Kurmi et al.^23^, the amino acids flanking the K302 succinylated lysines were enriched in nonpolar hydrophobic amino acids, such as leucine and isoleucine (Fig. 5e). We then analyzed the conservation of K302 and found that this site is highly conserved in MFF across various species (Fig. 5f).

We next confirmed whether CPT1A functions to succinylate MFF at K302. Flag-tagged wild-type (Flag-WT), K302R (Flag-K302R) and K302E (Flag-K302E) mutant MFF were transfected into SKOV-3 cells separately. As shown in Fig. 5g, both mutations decreased the succinylation levels of MFF, indicating that CPT1A can succinylate MFF at K302 and protect MFF from degradation. In addition, to further analyze the role of K302 succinylation in MFF protein stability, we checked the ubiquitination modification of Flag-tagged MFF WT, K302R and K302E which were exogenously expressed in SKOV-3 cells (Fig. 5g). Compared with WT, the K302R (mimic of deletion) mutation enhanced MFF ubiquitination, while K302E, a mimic of the negatively charged succinyl lysine modification, decreased MFF ubiquitination and protected MFF from degradation (Fig. 5g). In addition, 293TN cells transfected with WT, K302R or K302E MFF were treated with cycloheximide for up to 12 h. As shown in Fig. 5h, the K302E mutation significantly slowed the downregulation of MFF protein compared to the WT, while K302R decreased the half-life of MFF protein, indicating that succinylation of the K302 site protects MFF protein from ubiquitin-proteasome-mediated degradation.

To test whether the LSTase activity of CPT1A that stabilizes MFF may contribute to cell proliferation in ovarian cancer cells, we exogenously overexpressed H473A in the background of CPT1A knockdown and found that CPT1A WT rescued mitochondrial fission and cellular ATP production, but H473A did not (Fig. 5i, Supplementary Fig. 5c, d). As expected, H473A neither restored the inhibition of the mitochondrial oxygen consumption rate nor the inhibition of cell proliferation caused by CPT1A knockdown (Supplementary Fig. 5e–h), further suggesting that CPT1A regulates the stability of MFF through its LSTase activity and promotes the growth and proliferation of ovarian cancer cells.

### MFF might be a target for ovarian cancer treatment

**Fig. 6:**
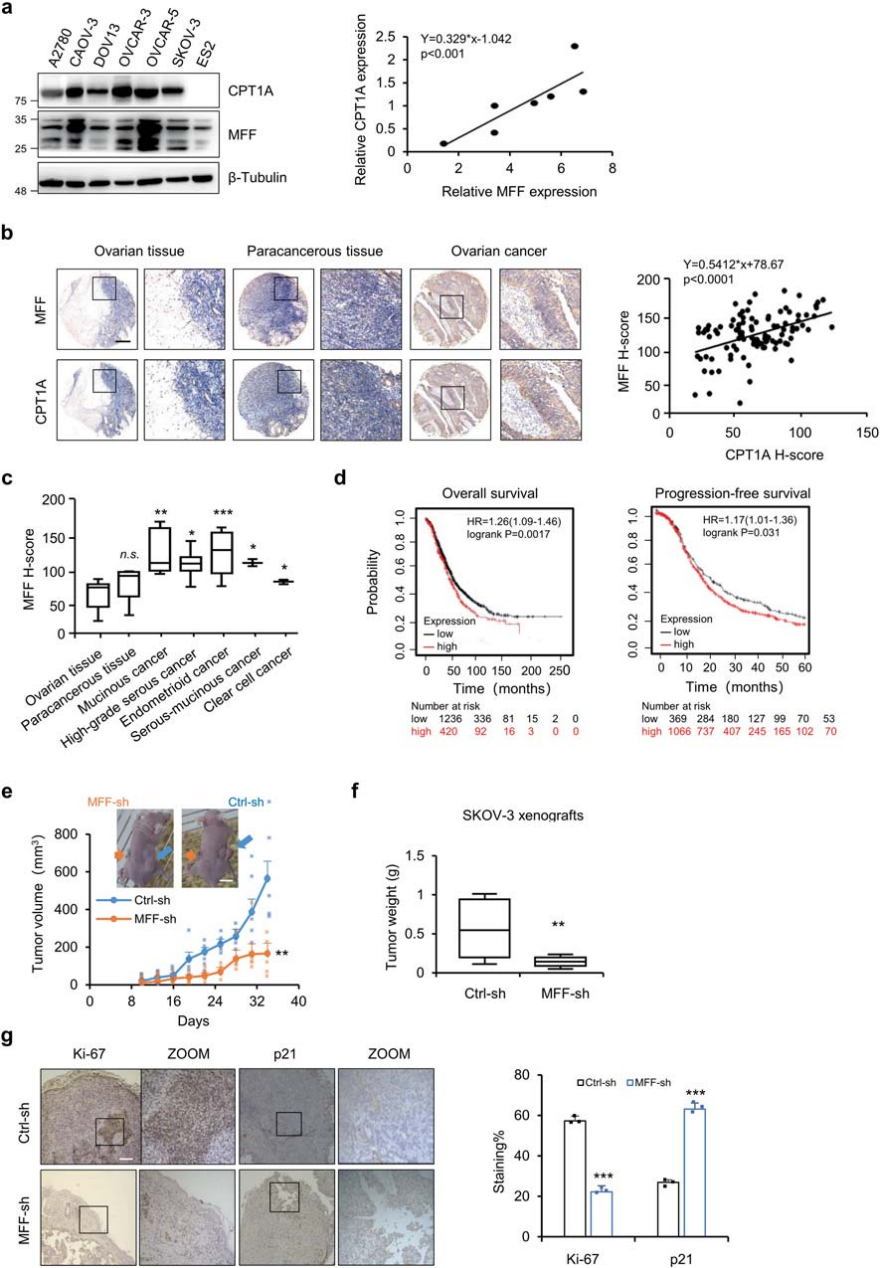
MFF might be a target for ovarian cancer treatment. **a** CPT1A and MFF was detected by WB in ovarian cancer cells (left). The WB bands density were quantified with ImageJ and normalized to β-tubulin. The expression relationship of CPT1A and MFF was analyzed (right). **b** CPT1A and MFF in tissue array were analyzed by IHC. Representatives images of ovarian cancer tissue, paracancerous tissue and normal ovarian tissue were shown (Scale bar = 200 μm, left). The expression relationship between CPT1A and MFF in tissue array was analyzed on the basis of H-score (right). **c** Based on H-score, the relative expression of MFF in normal ovarian tissues and various ovarian cancer and paracancerous tissues was analyzed (*n* = 100 cases). **d** Overall survival and progression-free survival rates were analyzed with Kaplan-Meier survival analysis for the relationship between survival time and MFF expression in ovarian cancer patients using the online tool (http://kmplot.com/analysis/). **e** MFF knockdown and control SKOV-3 cells were injected subcutaneously on the left and right flank of nude mice, respectively. Growth curves were prepared from tumor volumes measured at indicated times post cell injection (mean ± SD, *n* = 5 nude mice). Insets show MFF knockdown and control tumors. Scale bars, 12 mm. **f** At the end of the experiment, tumors were harvested and weighed, *n* = 5 nude mice. **g** Xenografts were sectioned and stained with anti-Ki-67 and anti-p21 antibodies (left). Quantitative analysis of Ki-67-positive and p21-positive cells staining area were performed with ImageJ (*n* = 3 for each group, right). Data represent the mean ± SD; *p < 0.05; **p < 0.01. ***p < 0.001, *n.s*. indicates no significant difference compared with the control groups or as indicated. Scale bars, 50 μm.

Our results suggest that CPT1A and MFF are positively correlated at the protein level due to posttranslational modification. We detected the protein expression of CPT1A and MFF in ovarian cancer cell lines and found that CPT1A and MFF were indeed positively correlated at the protein level (Fig. 6a). Given that the high expression of CPT1A is closely related to the onset and development of ovarian cancer and patient survival^27^, we further explored the correlation of MFF and clinical outcomes of ovarian cancer patients. A tissue array with 100 paraffin-embedded samples, including 80 ovarian cancer tissues, 10 paracancerous tissues, and 10 normal ovarian tissues, were stained with MFF or CPT1A antibody by immunohistochemistry (IHC). Similar to the ovarian cancer cell line results, the expression of MFF and CPT1A showed a high positive correlation (Fig. 6b). Meanwhile, the results also showed that, similar to CPT1A, MFF had a higher IHC score for protein expression in endometrioid and mucinous ovarian cancers (Fig. 6c). Furthermore, Kaplan-Meier survival analysis from the TCGA data showed that ovarian cancer patients with high MFF expression correlated with a significantly shorter overall survival (*p* = 0.0017) and a shorter Progression-free survival (*p* = 0.031) than those with low MFF expression (Fig. 6d). The results suggest that the expression of MFF in ovarian cancer patients correlates with poor clinical outcomes and that MFF could serve as an important prognostic marker.

To further examine the role of MFF in the onset and development of ovarian cancer, we next examined the effect of MFF knockdown on the tumorigenesis of SKOV-3 cells in nude mice. MFF knockdown strongly inhibited the initiation and development of subcutaneous xenografts in nude mice, as reflected by their growth curves and tumor weights (Fig. 6e, f). In addition, IHC staining showed that the positive rate of Ki-67 in tumor cells of the MFF knockdown group was significantly lower, meanwhile the expression of p21 was increased (Fig. 6g), indicating that MFF played a role in promoting carcinogenesis in ovarian cancer.

## Discussion

**Fig. 7:**
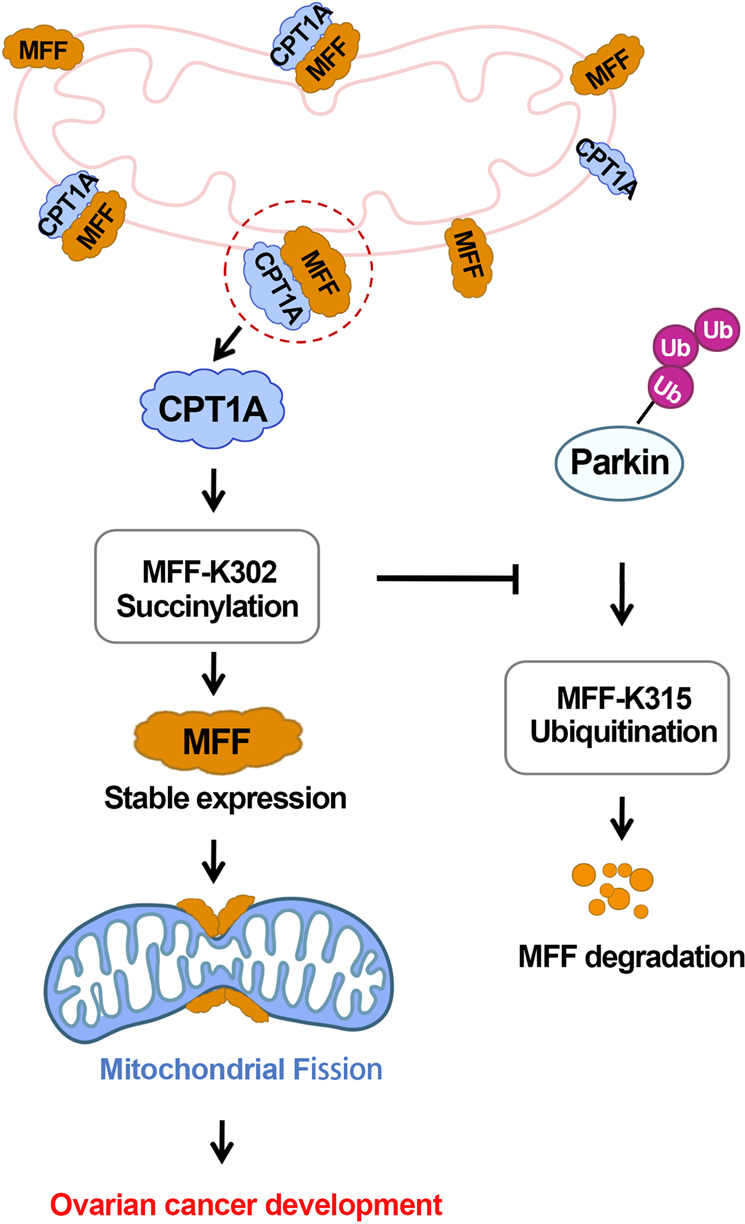
Schematic of CPT1A promoting MFF succinylation to regulate mitochondrial dynamics and promote the development of ovarian cancer. In ovarian cancer cells, CPT1A functions as a succinyltransferase to promote MFF succinylation at the lysine 302 (K302), which prevents Parkin-mediated ubiquitin-proteosome degradation of MFF at lysine 315 (K315), thus stabilizing the expression of MFF. The highly expressed MFF enhances mitochondrial fission, which in turn promotes the progression of ovarian cancer.

Mitochondrial dynamics are critical for regulating cellular homeostasis and survival. Disruption or imbalance in mitochondrial dynamics can lead to mitochondrial dysfunction, which in turn leads to various human diseases ranging from neurodegenerative diseases to cancer. Therefore, proteins that control mitochondrial dynamics are considered important regulators of mitochondrial function and mitochondrial quality control in health and disease. In this study, we found that CPT1A promoted MFF succinylation through the action of its lysine succinylase, thereby interfering with its recognition and binding to Parkin and inhibiting the ubiquitination and degradation of MFF. The high expression of MFF in ovarian cancer promotes mitochondrial fission and enhances mitochondrial function, thereby promoting the development of ovarian cancer (Fig. 7).

The balance of mitochondrial dynamics is controlled by the presence of fusion-promoting or fission-promoting macromolecules. How these macromolecules are regulated is the key to studying abnormal mitochondrial dynamics. Here, we showed evidence that CPT1A could regulate the stability of MFF at the posttranslational modification level, thereby promoting the fission state of mitochondria in ovarian cancer. As a constituent protein in the mitochondrial outer membrane, the expression of CPT1A is closely related to the shape and function of mitochondria. Luo et al. found that CPT1A overexpression increased the phosphorylation of DRP1 at Ser637 to promote mitochondrial fusion and inhibit glioblastoma stem cell self-renewal^36^. Abnormal mitochondrial structure and dysfunction are characteristics of kidney disease pathogenesis^37^. CPT1A overexpression promotes mitochondrial biogenesis and prevents mitochondrial dysfunction^20^. Using a mouse ovarian surface epithelial model, Grieco et al. found that mitochondrial morphology changes from a filamentous network to a single enlarged organelle with increasing malignancy in serous ovarian cancer^8^. In view of the high expression of CPT1A in ovarian cancer tissue cells compared with normal tissue cells^27^, our work suggests that the enhancement of mitochondrial fission in ovarian cancer tissue cells may be related to the high expression of CPT1A, and that CPT1A regulates mitochondrial dynamics.

As a key rate-limiting enzyme in mitochondrial long-chain free fatty acid uptake for beta-oxidation (FAO), CPT1A plays an important role in tumor progression, epithelial-mesenchymal transition (EMT) and migration^38^. It has also been reported to play an important role in the stemness maintenance and differentiation of embryonic brain neural stem cells and adult neural stem cells^39,40^. These studies are based on the function of CPT1A-mediated FAO. Recently, studies have found that CPT1A itself has lysine succinyltransferase activity^23^. CPT1A regulates the expression and stability of its substrate proteins, such as S100A10 and enolase 1, through CPTase-independent lysine succinyltransferase activity, thereby promoting tumor cell growth, proliferation and migration^25^. Here, we report the novel finding that MFF is a substrate for CPT1A LSTase activity. Our results further confirmed that CPT1A has LSTase activity independent of its CPTase activity and revealed a new modification and regulation mode of MFF through which CPT1A regulates mitochondrial morphology.

Under normal physiological conditions, mitochondrial fission leads to two outcomes: either the biogenesis of new mitochondria or the removal of dysfunctional mitochondria through mitophagy. Studies have shown that, unlike Fis1, MFF promotes mitochondrial fission mainly in the middle of the mitochondria, thereby promoting mitochondrial biogenesis and ultimately enhancing mitochondrial function and cellular ATP^41^. In this study, the high expression of CPT1A might not promote mitochondrial biogenesis but might maintain mitochondrial fission to promote mitochondrial function through MFF, which is beneficial to the rapid metabolism of tumor cells. After knockdown of CPT1A or MFF, mitochondrial fusion is enhanced, and tumor cell growth is inhibited, partly because fused mitochondria might lead to the accumulation of damaged or senescent mitochondria in cells, which is not conducive to cell survival.

Imbalances in mitochondrial dynamics, with enhanced fission or reduced fusion, have been found in patients with a variety of tumors and lead to morphological fragmentation of mitochondria. Increased expression of fission-related proteins or decreased expression of fusion-related proteins has been found in various tumors, such as liver cancer^42^, breast cancer^43^, and lung cancer^6^, suggesting that these mitochondrial dynamics-related factors may be targets for tumor therapy. In this study, we confirmed that MFF is highly expressed in ovarian cancer cells. Knockdown of MFF significantly inhibited the growth and proliferation of ovarian cancer cells in vitro and in vivo, indicating that MFF can be a therapeutic target for ovarian cancer therapy.

## Methods

### Cell cultures

Human SKOV-3, A2780, OVCAR-3, OVCAR-5, CAOV-3, DOV13, ES2 and 293TN cells were cultured in RPMI-1640 (Solarbio, Beijing, China) or DMEM (Gibco, Thermo Fisher Scientific, US) accordingly, supplemented with 10% FBS (Biological Industries, Shanghai, China) and 1% penicillin-streptomycin (Solarbio) and were incubated in a humidified incubator at 37 °C containing 5% CO_2_. The SKOV-3, A2780, OVCAR-3, CAOV-3 and ES2 cells obtained from ATCC. The OVCAR-5 cell purchased from Shanghai Qincheng Biotechnology Co., Ltd. The DOV13 cell was provided generously by Dr Fang (Virginia Commonwealth University). And 293TN were obtained from System Biosciences (SBI, CA, USA). All the cells were confirmed by a vendor via short tandem repeat profiling and were not reauthenticated by the authors. The cell lines were expanded at low passages and stored in liquid nitrogen after receipt. Cells were used within ten passages for experiments or resuscitated within 1 month.

### Plasmid construction and lentivirus packaging

For shRNA knockdown, shRNA lentivirus vectors were generated by cloning target-specific oligonucleotides into the pGreenPuro shRNA plasmid according to the manufacturer’s protocol (SBI, CA, USA). The target sequences of the shRNA template oligonucleotide (5’-3’) were as follows: CPT1A-sh1: GGGAGTACGTCATGTCCATTG; CPT1A-sh2: GCGTTCTTCGTGACGTTAGAT; MFF-sh: AACGCTGACCTGGAACAAGGA; MFN2-sh: GGAAGAGCACCGTGATCAATG; Parkin-sh: GCCTTCTGCCGGGAATGTAAA; March5-sh: GGTTTACGTCTTGGATCTTGC. For exogenous overexpression, CPT1A and MFF (transcript 2) were cloned from SKOV-3 cDNA using Max DNA (TaKaRa, Dalian, China) according to the manufacturer’s instructions. The mutants CPT1A-G710E, CPT1A-H473A and MFF-K315R were generated using QuikChange mutagenesis. The WT and mutant CPT1A or MFF were subcloned into the pCDH-3✕Flag-CMV vector to create Flag-tagged expression plasmids. All the constructs were verified by sequencing. The lentiviruses were produced by cotransfection of 293TN cells with a lentiviral vector, psPAX2 and pMD2.G (Addgene, USA) using Lipofectamine 2000 (Life Technology, Thermo Fisher Scientific, MA, USA) as previously described^44^. The virus-containing supernatants were harvested at 48 and 72 h posttransfection and were used for infection.

### Western blotting, antibodies and immunohistochemistry (IHC) staining

Whole-cell lysates were prepared with cell lysis buffer (20 mM Tris-HCl (pH 7.5), 150 mM NaCl, 1% Triton X-100, 1 mM PMSF, 1 mM Na_3_VO_4_, and 1× proteinase inhibitor (Roche, Basel, Switzerland)). The protein concentration was determined using the BCA protein assay kit (Solarbio, Beijing, China). Equal amounts of protein were separated by SDS-PAGE and were transferred to polyvinylidene difluoride (PVDF) membranes (IPVH 00010, Millipore, MA, USA). The membranes were blocked and then probed with primary antibodies overnight at 4 °C. Beta-Tubulin was used as an endogenous loading control. The membranes were incubated with a horseradish peroxidase-conjugated secondary antibody (1:2000, Cell Signaling, Beverly, MA, USA) and visualized by ECL chemiluminescence (Millipore, MA, USA). Western blotting bands were quantified and statistically analyzed using ImageJ. Xenograft tumors were fixed with 10% neutral buffered formalin overnight and embedded in paraffin (FFPE). FFPE blocks were cut at 5 µm thickness, dried in a 60 °C oven overnight and stained with hematoxylin and eosin and antibodies (anti-Ki67 1:500, anti-p21 1:200). Ovarian cancer patient tissue microarrays were obtained from Avilabio (#DC-Ova11039, Avilabio Biotechnology, China) and stained with anti-CPT1A or anti-MFF antibodies. Each of the IHC-stained sections was scanned using a Pannoramic Digital Slide Scanner (Pannoramic MIDI, 3D HISTECH) and scored according to the percentage of immunostaining and the staining intensity (0, negative; 1+, weak; 2+, moderate; and 3+, strong) as described previously^45,46^. An H-score (histochemistry score) was calculated using the following formula: H-score = (percentage of weak intensity area ×1) + (percentage of moderate intensity area ×2) + (percentage of strong intensity area ×3). The Antibodies used for western blotting and IHC staining are shown in Supplementary Table 1.

### Immunofluorescence and confocal microscopy

The xenografts (ctrl-sh, MFF-sh or CPT1A-sh2) were embedded in paraffin, sectioned, dehydrated, subjected to antigen retrieval, blocked with 1% BSA for 30 min, and incubated with TOM20 (#13929S, CST) overnight at 4 °C. After washing in 0.1% BSA in PBS, paraffin sections were incubated with secondary antibodies conjugated with Alexa Fluor 594 (Molecular Probes, OR, USA). Cell nuclei were visualized with DAPI. Images were acquired by a confocal laser-scanning microscope (Leica, IL, USA). For the colocalization of DRP1 and mitochondrion, SKOV-3 cells seeded on coverslips were incubated with 50 nM MitoTracker (#9082, CST, USA) for 30 min. After washing in PBS, the cells were fixed in cold methanol and then blocked and incubated with primary antibodies (DRP1, 1:200) overnight. After washes in 0.1% BSA in PBS, cells were incubated with secondary antibodies conjugated with Alexa Fluor 488 (Molecular Probes, OR, USA). Cell nuclei were visualized with DAPI. Images were acquired by a confocal laser-scanning microscope (Leica, IL). For mitochondrial morphology analysis, cells expressing DsRed-mito (Miaoling, Wuhan, China) were infected with lentivirus expressing shRNA or exogenous overexpression plasmids of CPT1A, MFF, MFN2, or Parkin. The infected cells were imaged using a confocal laser-scanning microscope (Leica, IL, USA).

### Live imaging of mitochondria

Cells expressing DsRed-mito (Miaoling, Wuhan, China) were infected with lentivirus expressing shRNA or exogenous overexpression plasmids of CPT1A, MFF, MFN2, or Parkin and plated in glass chamber slides (24-well glass-bottom Petri dishes, #801006 NEST, China). Imaging was started immediately at 37 °C and 5% CO_2_. Images were acquired by DeltaVision microscopes (General Electric Company, USA).

### Real-time PCR

Real-time PCR analysis was performed as previously described^44^. Briefly, total RNA was extracted from SKOV-3 or OVCAR-3 cells with a total RNA Simple Kit (GenStar, Beijing, China). First-strand cDNA was synthesized with Max DNA (TaKaRa, Dalian, China) according to the manufacturer’s instructions. Fluorogenic probes were purchased from TaKaRa (#RR820A, Shiga, Japan). The relative mRNA levels of Parkin, March5 and MFF were quantified using a real-time PCR reaction platform (CFX96 Touch, Bio-Rad, MA, USA). The specific primer sets used for this assay were shown as follows (5′-3′): CPT1A (forward: ATCAATCGGACTCTGGAAACGG, reverse: TCAGGGAGTAGCGCATGGT), MFF (forward: CGGAGAGGATTGTTGTAGCAG, reverse: TGGTCTTTCACTCAGCGTAAG), Parkin (forward: CGGGAAAACTCAGGGTACAG, reverse: AAATTCTGCACTAGTCCCAGG), March5 (forward: GGGTGGATGAAAAGCAAAGAG, reverse: TGGACATGCCTTTGAGATCAG) and GAPDH (forward: GGGAAGGTGAAGGTCGGA, reverse: GCAGCCCTGGTGACCAG). GAPDH was used as an internal control for normalization.

### Cell growth count, colony formation and EdU cell proliferation assay

SKOV-3 (1 × 10^4^) and OVCAR-3 (1 × 10^4^) cells seeded into 24-well plates were cultured for cell growth and proliferation analysis. Cells were harvested and stained with trypan blue, and live cells were counted.

For colony formation assays, 500 SKOV-3 cells/well and 1000 OVCAR-3 cells/well were seeded into 12-well plates for 2–3 weeks. The cell culture media were replaced every 3–4 days. The cells were fixed with 4% paraformaldehyde for 30 min, stained with crystal violet for 30 min after 2–3 weeks and enumerated by using ImageJ. ImageJ filters scored colonies that were 100 µm (SKOV-3) or 50–100 µm (OVCAR-3) in size.

EdU cell proliferation assays were performed by using the BeyoClickTM EdU Cell Proliferation Kit according to the manufacturer’s protocol (#C0075L, Beyotime, Shanghai, China). Briefly, SKOV-3 and OVCAR-3 cells seeded on coverslips were incubated with EdU (10 μM) at 37 °C for 2 h. After labeling, cells were fixed, permeabilized, and then incubated with click reaction solution (containing Alexa Fluor 555, Red). Cells were then counterstained with Hoechst 33342 (blue). Images were acquired with a fluorescence microscope (Axio Imager. M2, GER).

### Transmission electron microscopy (TEM)

Cells were fixed with 2.5% glutaraldehyde for 2 h at 4 °C. After being washed in 0.1 M PB three times and fixed in 1% osmic acid, the cells were washed with ddH_2_O and then dehydrated in 50, 70, 90, and 100% ethanol for 15 min each. After being embedded in a gradient propylene oxide and resin series, the samples were further embedded in Embed 812 resin. After cutting into 60 nm ultrathin sections, the samples were counterstained with uranyl acetate and lead citrate. Images were acquired via a HITACHI 7700 TEM (Tokyo, Japan).

### ATP quantification

ATP content was measured using a firefly luciferase-based ATP assay kit (S0026, Beyotime) according to the manufacturer’s instructions. In brief, cells were lysed in 200 μl of lysis buffer/3 cm dish and collected for centrifugation at 12,000 × *g* for 5 min at 4 °C. The supernatants were collected. Two microliters were used for protein concentration measurement, and the remnant was incubated with the luciferin substrate and luciferase enzyme in the dark for 1 min to stabilize the luminescent signal. A fluorescence microplate reader was used to measure the bioluminescence intensity. The ATP concentration was calculated from the standard curve.

### Xenograft study

The experimental protocols were approved and performed in accordance with the ethical principles and guidelines formulated by the Animal Care and Use Committee of Shaanxi Normal University. BALB/c nude mice (female), 5–6 weeks old, were purchased from Beijing HFK Bioscience Co., Ltd. and used for experiments. Control, MFF-knockdown or CPT1A-knockdown SKOV-3 cells (5 × 10^6^) were subcutaneously implanted into the right or left flank of nude mice. Nude mice body weight was also recorded three times weekly, and the mice were observed daily. As previously described the tumor volumes were calculated using the following formula: volume (mm^3^) = 0.5 × longest tumor diameter × (shortest tumor diameter)^2^ ^44^. At the endpoint, the mice were euthanized and tumors were harvested for weight and immunohistochemistry staining analysis.

### LC-MS/MS Analysis

LC-MS/MS analysis was performed by Hoogen Biotechnology as described^47^. Briefly, SKOV-3 cells were harvested and MFF was enriched by immunoprecipitation with an anti-MFF antibody. After separation by SDS-PAGE, the MFF region was cut out digested, and separated using the EASY-nLC 1000 ultrahigh performance liquid phase system. The resulting MS/MS data were processed using Proteome Discoverer 2.1. Tandem mass spectra were searched against the NCBI human MFF sequence. The search parameters as follows: the digestion protease was trypsin; modification of +114 Da on Lys was considered as ubiquitination, and modification of +100 Da on Lys was considered as succinylation. The mass error was set to 10 ppm for precursor ions and 0.02 Da for fragment ions. Peptide confidence was set at high, and the peptide ion score was set >20.

### Mitochondrial stress assay

The oxygen consumption rate (OCR) was measured using a Seahorse XF8 analyzer and an XF assay kit (Agilent Technologies). Cells were seeded at 2 × 10^5^ cells/well on an XF8 plate for 24 h before the assay. On the day of the assay, the medium was changed to XF Base Medium (0 mM glucose, Agilent Technologies) supplemented with 10 μM L-glutamine, 200 μM glucose, 10 μM sodium pyruvate without serum. Then, the plates were incubated for 1 h in a non-CO_2_ incubator at 37 °C. The reagents for the assay were prepared using an XF Cell Mito Stress Test kit (Agilent Technologies) according to the manufacturer’s instructions, and injection was performed according to a standard assay protocol (Port A: oligomycin; Port B: FCCP; and Port C: rotenone/antimycin A).

### Statistics and reproducibility

All experiments were performed at least three times, and results were presented as the means ± SD. To determine the significance between the tested groups, Student’s *t* test or GraphPad Prism 7 was used, where *p* values <0.05 were considered as statistically significant.

### Reporting summary

Further information on research design is available in the Nature Portfolio Reporting Summary linked to this article.

## Supplementary information

Supplementary Information

Description of Additional Supplementary Files

Supplementary Data 1

Supplementary Data 2

Reporting Summary

## Data Availability

The mass spectrometry proteomics data have been deposited to the ProteomeXchange Consortium via the PRIDE partner repository with the dataset identifier PXD041990. The source data for graphs are provided in Supplementary data 1. The ovarian cancer patient tissue microarrays details are uploaded in Supplementary data 2. Uncropped/unedited blots are provided in the Supplementary Information as Supplementary Fig. 6. All data generated or analyzed during this study are available from the corresponding author on reasonable request.
