# Supplementary material for: Carnitine palmitoyltransferase 1A promotes mitochondrial fission by enhancing MFF succinylation in ovarian cancer
Source: Commun Biol. 2023 Jun 8;6:618. doi: 10.1038/s42003-023-04993-x (PMC10250469; doi:10.1038/s42003-023-04993-x)
Supplement: Supplementary file 1 — Supplementary Information [file 42003_2023_4993_MOESM1_ESM.pdf]

Supplementary Fig. 1

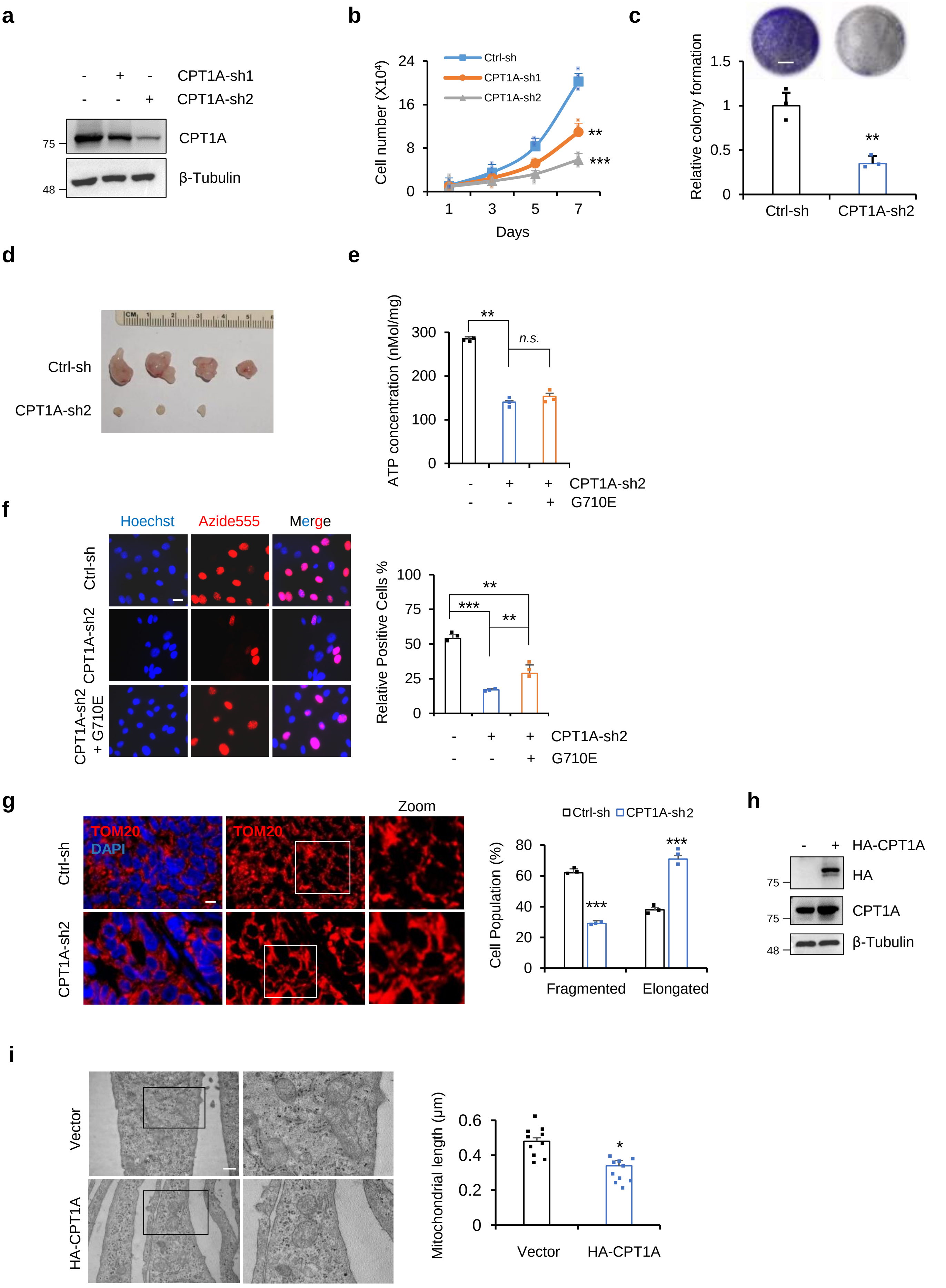

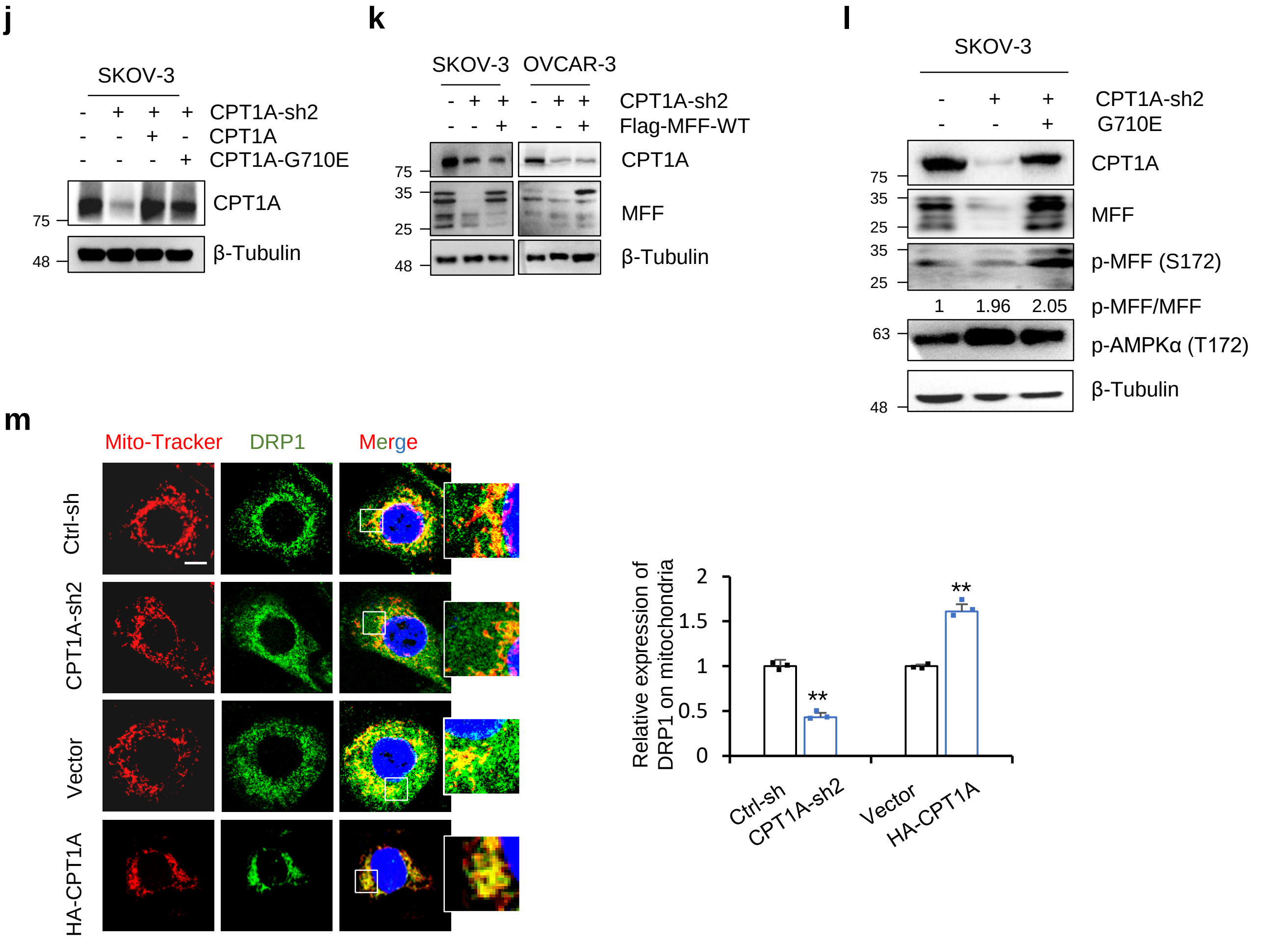

Supplementary Fig. 1. CPT1A knockdown significantly inhibited the growth of ovarian cancer cells.

a CPT1A was knocked down by shRNA in SKOV-3 cells. The CPT1A expression was examined by western blotting. **b** Growth curves of SKOV-3 cells with control or CPT1A knockdown, seeded in 24-well plates, were constructed from counting for up to 7 days, n=3 for each group. **c** Colony formation assay was performed in SKOV-3 cells with control and CPT1A knockdown. Microphotographs covering representative areas of each treatment were shown (upper). Numbers of colonies in each well of six-well plates were analyzed (n=3 for each group, lower). Scale bar = 10 mm. **d** SKOV-3 cells (5×10^6) with control or CPT1A knockdown were injected subcutaneously on the right flank of nude mice. Xenograft tumors were harvested 3 weeks after inoculation (n=4 nude mice). **e** ATP concentration was detected in SKOV-3 cells with control, CPT1A knockdown and CPT1A-G710E (G710E) overexpression in conjunction with CPT1A knockdown, n=3 for each group. **f** The EdU proliferation assay was performed in SKOV-3 cells with control, CPT1A knockdown or exogenous overexpression of G710E in conjunction with CPT1A knockdown. Representative image (left) and the ratio of EdU-positive cells (n=3 for each group, right) are shown. Scale bar = 25 μm. **g** Immunofluorescent staining of TOM20 in xenograft tumor cells revealed that CPT1A knockdown promotes mitochondrial fusion *in vivo* (left, scale bar=10 μm). The proportion of cells (n=3 samples, each sample contains 100 cells) with fragmented, or elongated mitochondria was quantified (right). **h** Western blotting analysis of HA-tagged CPT1A exogenous overexpression in SKOV-3 cells. **i** Representative images showing mitochondrial morphology in SKOV-3 cells with empty vector or HA-CPT1A by transmission electron microscopy (left, scale bars=500 nm). Quantification of mitochondrial morphology in electron microscope images (n=10 for each group, right).

j Western blotting analysis of CPT1A in SKOV-3 cells with Ctrl-sh, CPT1A knockdown, exogenously overexpressed CPT1A-WT + CPT1A-sh and CPT1A-G701E + CPT1A-sh. **k** Western blotting analysis of CPT1A and MFF in SKOV-3 and OVCAR-3 cells with exogenously overexpressed MFF in conjunction with CPT1A knockdown. **l** MFF, p-MFF (S172) and p-AMPKα (T172) were analyzed by western blotting in SKOV-3 cells with control, CPT1A knockdown and G710E overexpression in conjunction with CPT1A knockdown. The relative p-MFF expression was normalized to MFF and then compared to control groups. **m** Detection of DRP1 (green), MitoTracker (red) by immunoflfluorescence in SKOV-3 cells. Representative imagines are shown (left). The relative colocalization of DRP1 with mitochondria was quantified by using imageJ (right, n=3 for each group). scale bar=10 μm. Data represent the mean ± SD; *p < 0.05, **p < 0.01 and ***p < 0.001, *n.s.* indicates no significant difference compared with the control groups.

Supplementary Fig. 2

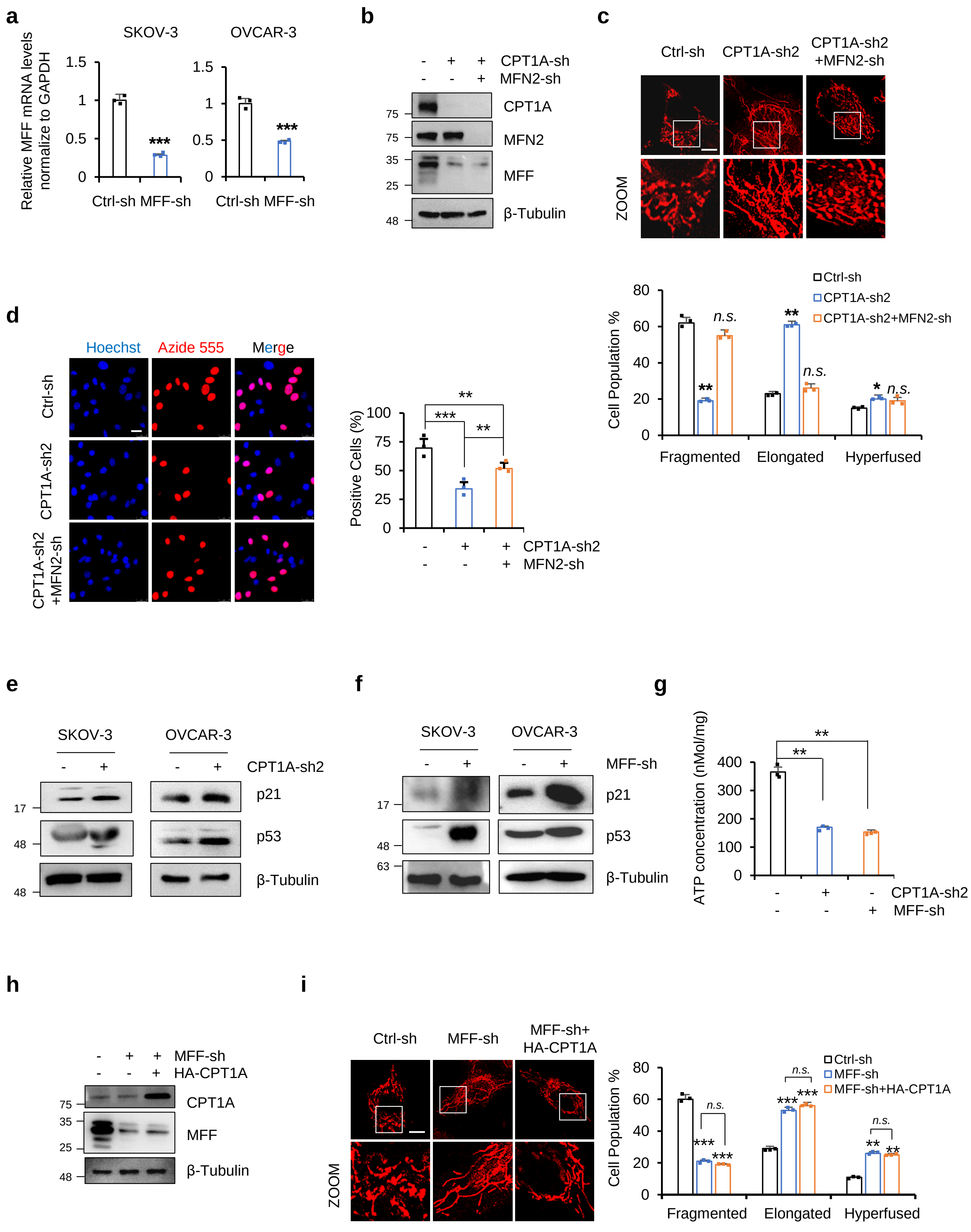

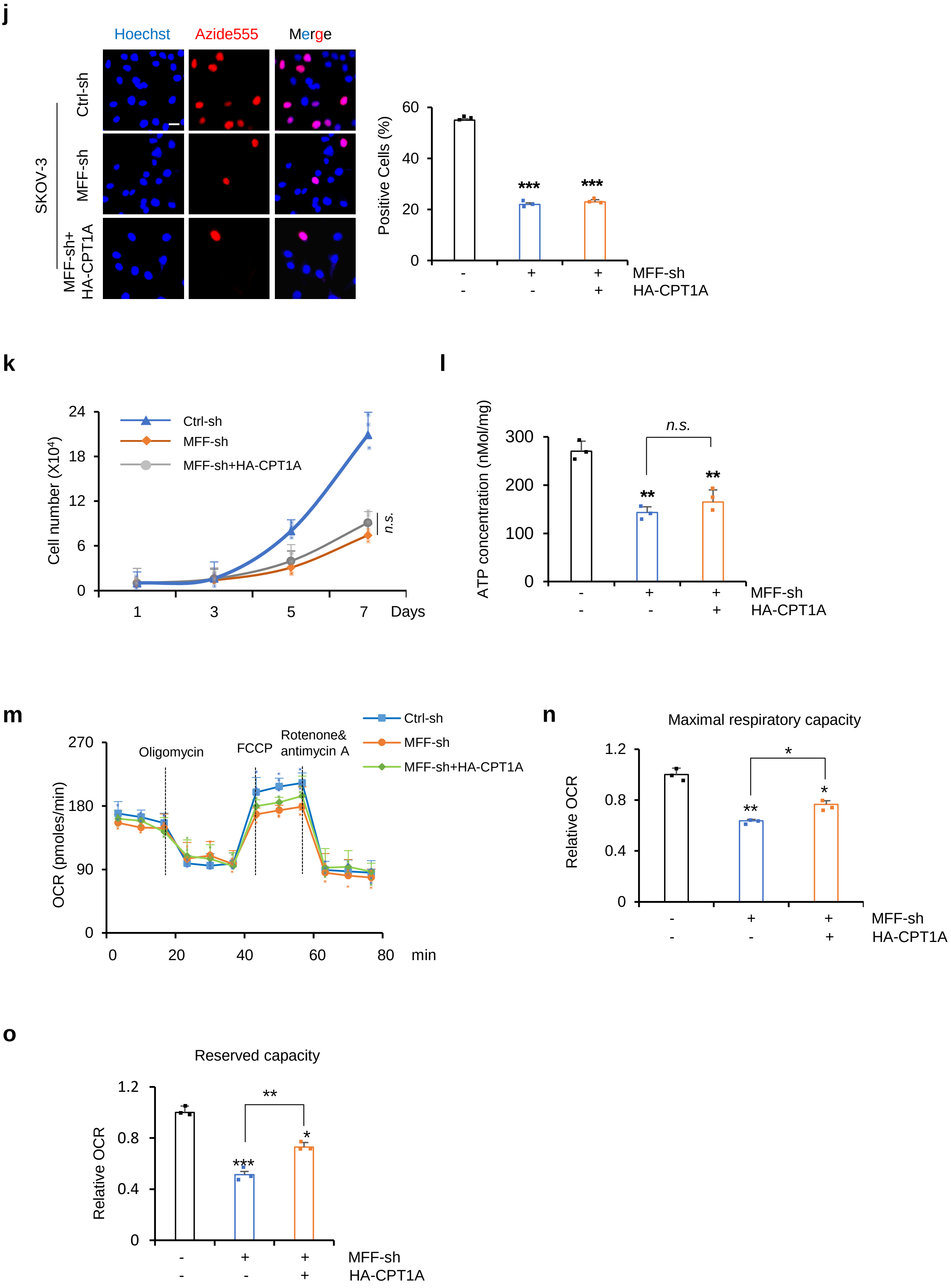
j
Hoechst
Azide555
Merge
SKOV-3
Ctrl-sh
MFF-sh
MFF-sh+
HA-CPT1A
Positive Cells (%)
60
40
20
0
***
***
-
+
+
MFF-sh
-
-
+
HA-CPT1A
k
Ctrl-sh
MFF-sh
MFF-sh+HA-CPT1A
Cell number (X10^4)
24
18
12
6
0
n.s.
1
3
5
7
Days
l
ATP concentration (nMol/mg)
300
200
100
0
n.s.
**
**
-
+
+
MFF-sh
-
-
+
HA-CPT1A
m
Ctrl-sh
MFF-sh
MFF-sh+HA-CPT1A
OCR (pmoles/min)
270
180
90
0
Oligomycin
FCCP
Rotenone&
antimycin A
0
20
40
60
80
min
n
Maximal respiratory capacity
Relative OCR
1.2
0.8
0.4
0
*
**
*
-
+
+
MFF-sh
-
-
+
HA-CPT1A
o
Reserved capacity
Relative OCR
1.2
0.8
0.4
0
**
***
*
-
+
+
MFF-sh
-
-
+
HA-CPT1A

Supplementary Fig. 2. Mitochondrial dynamics affect the proliferation of ovarian cancer cells.

a The knockdown efficiency of MFF was examined in SKOV-3 and OVCAR-3 cells by real-time PCR, n=3 for each group. **b** Western blotting analysis of CPT1A, MFF and MFN2 in SKOV-3 cells with control, CPT1A knockdown and MFN2 knockdown in conjunction with CPT1A-sh. **c** Representative mitochondrial morphologies of SKOV-3 cells with control, CPT1A knockdown or CPT1A and MFN2 double knockdown. (upper, scale bar=10 μm). The proportion of cells with different mitochondrial morphologies was quantified (n=3 samples, each sample contains 100 cells, lower). **d** Cell proliferation assay was performed by EdU assy in SKOV-3 cell with control, CPT1A knockdown or CPT1A and MFN2 double knockdown, n=3 for each group, scale bar = 25 μm. **e** Induction of p21 and p53 by CPT1A knockdown was confirmed by immunoblotting. **f** Induction of p21 and p53 by MFF knockdown was confirmed by immunoblotting. **g** ATP concentration was analyzed in SKOV-3 cells with control, CPT1A knockdown and MFF knockdown, n=3 for each group. **h** CPT1A and MFF were examined by western blotting analysis in SKOV-3 cells with control, MFF knockdown and exogenous overexpression of HA-CPT1A in conjunction with MFF knockdown. **i** Representative images of the mitochondrial morphology in SKOV-3 cells with control, MFF knockdown or exogenous overexpression of CPT1A-WT in conjunction with MFF knockdown (left, scale bar=10 μm). The proportion of cells (n=3 samples, each sample contains 100 cells) with fragmented, elongated and hyperfused mitochondria was quantified (right). **j** Cell proliferation assay was performed by EdU assay in SKOV-3 cell with control, MFF knockdown or exogenous overexpression of HA-CPT1A in conjunction with MFF knockdown, n=3 for each group. Scale bar = 25 μm. **k** Growth curves of SKOV-3 cells with ctrl-sh, MFF-sh and MFF-sh + HA-CPT1A, plated in 24-well plates, were constructed from quantification of cell numbers for up to 7 days, n=3 for each group. **l** ATP concentration was assessed in SKOV-3 cells with control, MFF knockdown and exogenous overexpression of HA-CPT1A in conjunction with MFF knockdown, n=3 for each group. **m** The oxygen consumption rate (OCR) assay was performed in in SKOV-3 cells with control, MFF knockdown and exogenous overexpression of HA-CPT1A in conjunction with MFF knockdown, n=3 for each group. **n** The quantification (n=3 for each group) of maximal respiratory capacity in **m**. **o** The quantification (n=3 for each group) of reserved capacity in **m**. Data represent the mean ± SD; *p < 0.05, **p < 0.01 and ***p < 0.001, as compared with control groups.

Supplementary Fig. 3

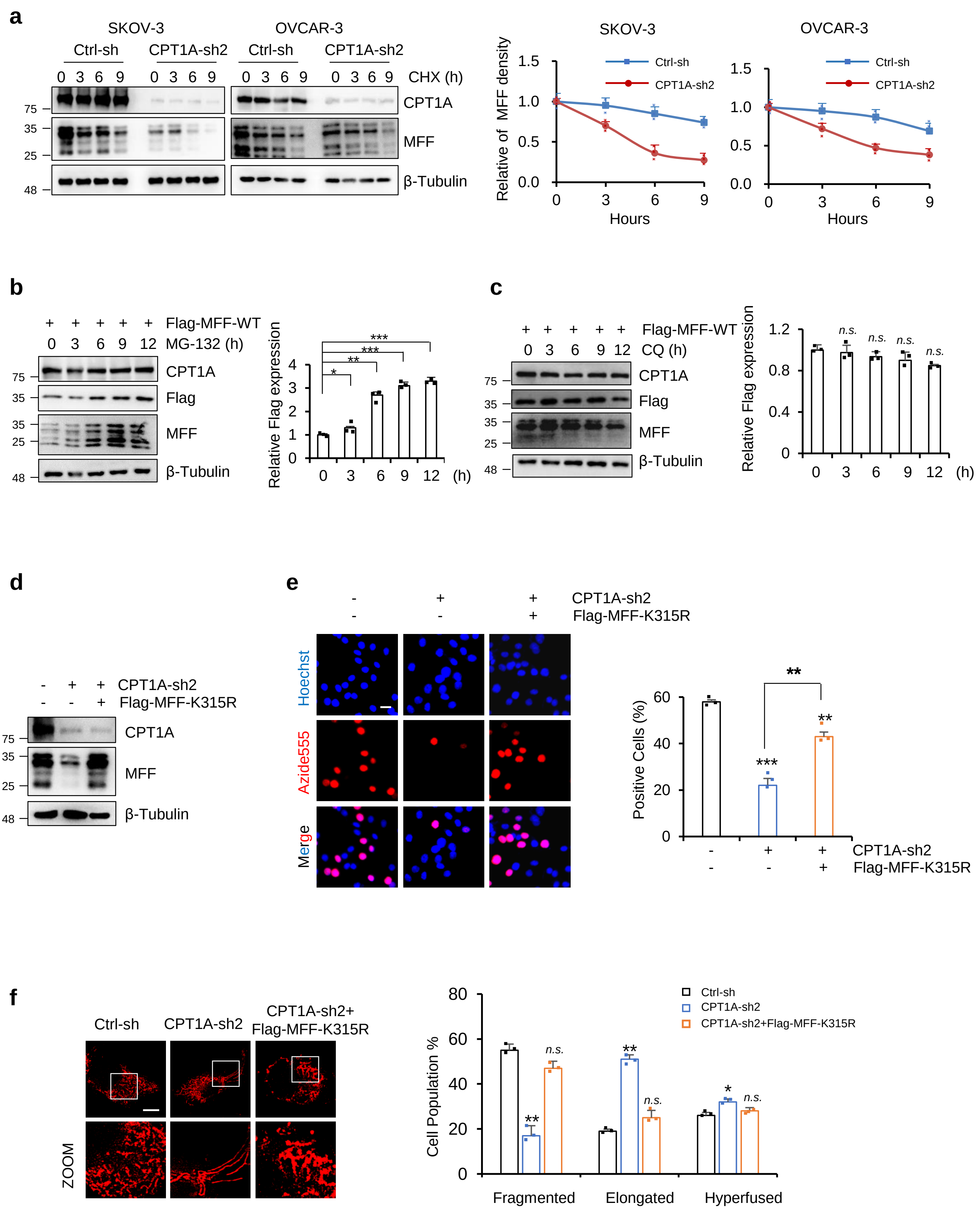

Supplementary Fig. 3. CPT1A knockdown leads to MFF degradation.

a SKOV-3 and OVCAR-3 cells with or without CPT1A-sh2 were treated with 10 μM cycloheximide (CHX) for up to 9 hours. Whole cell lysates were prepared and detected against CPT1A and MFF antibodies (left). And densitometric analysis of MFF immunoblot band was performed with ImageJ (n=3 for each group, right). **b, c** SKOV-3 (**b**) and ES2 (**c**) cells transfected with Flag-MFF-WT plasmid were treated with 10 μM cycloheximide (CHX) for up to 12 hours. Cells were harvested at the indicated time point and the expression of CPT1A and Flag-MFF were analyzed by western blotting (left). And densitometry of Flag immunoblot was performed with ImageJ and normalized to their β-tubulin. The relative expression of Flag-MFF was presented (n=3 for each group, right). **d** CPT1A and MFF was examined by western blotting analysis in SKOV-3 cells with control, CPT1A knockdown and exogenous overexpression of Flag-MFF-K315R in conjunction with CPT1A knockdown. **e** Cell proliferation assay was performed by EdU assay in SKOV-3 cell with control, CPT1A knockdown or exogenous overexpression of Flag-MFF-K315R in conjunction with CPT1A knockdown, n=3 for each group. Scale bar=25 μm. **f** Representative images of the mitochondrial morphology in SKOV-3 cells with control, CPT1A knockdown and exogenous overexpression of Flag-MFF-K315R in conjunction with CPT1A knockdown (left, scale bar=10 μm). The proportion of cells (n=3 samples, each sample contains 100 cells) with fragmented, elongated, and hyperfused mitochondria was quantified (right). Data represent the mean ± SD; $^{*}p < 0.05$, $^{**}p < 0.01$ and $^{***}p < 0.001$, *n.s.* indicates no significant difference compared with the control groups.

Supplementary Fig. 4

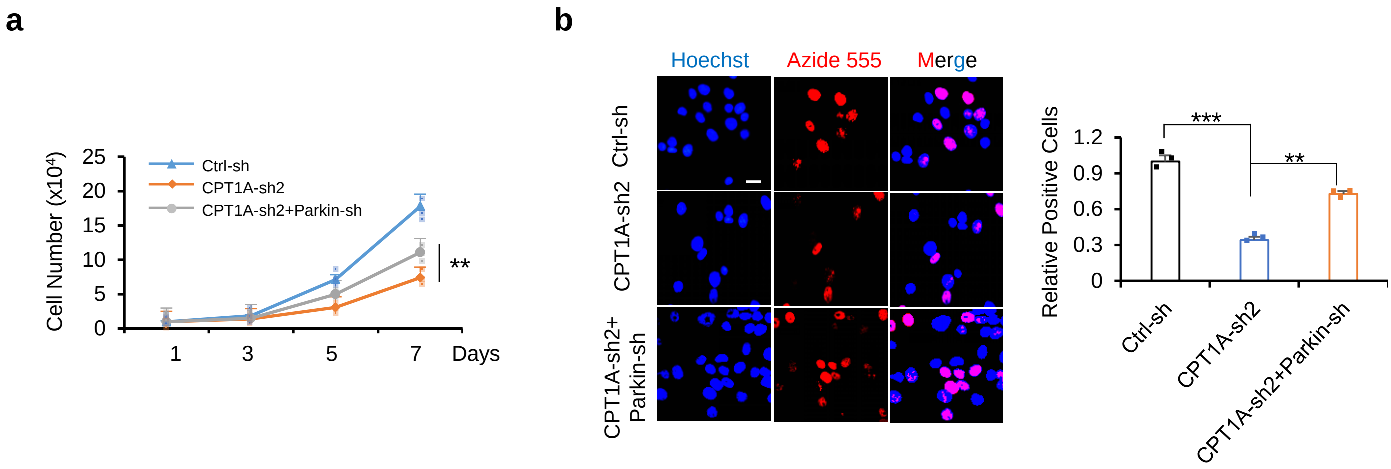

Supplementary Fig. 4. Parkin affects the proliferation of ovarian cancer cells.

a SKOV-3 cells with control, CPT1A knockdown, or CPT1A and Parkin double knockdown were seeded in 24-well plates. Growth curves were constructed from quantification of cell numbers for up to 7 days, n=3 for each group. **b** The EdU proliferation assay was performed in SKOV-3 cells with control, CPT1A knockdown, or CPT1A and Parkin double knockdown. Representative image (left) and the ratio of EdU positive cells (n=3 for each group, right) are shown. Scale bars=25 μm. Data represent the mean ± SD; $*p < 0.05$, $**p < 0.01$ and $***p < 0.001$, as compared with control groups.

Supplementary Fig. 5

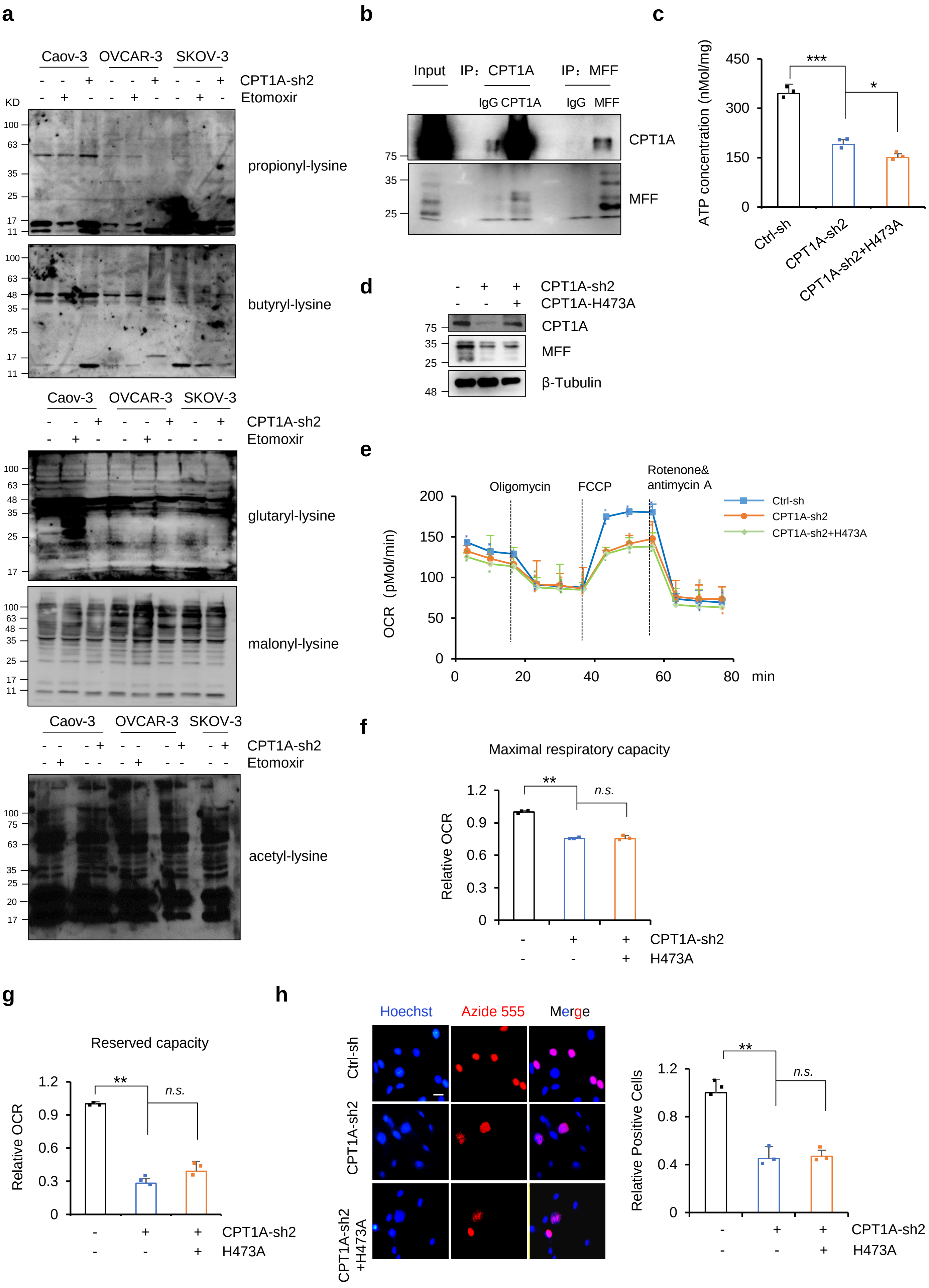

Supplementary Fig. 5. CPT1A promotes MFF succinylation.

a CAOV-3, OVCAR-3 and SKOV-3 cells with CPT1A knockdown or cells treated with etomoxir (10 μM) for 24 hours were harvested for acylation modifications by western blotting. Bands are derived from the different gels. **b** SKOV-3 cells were collected, co-immunoprecipitated with anti-CPT1A and anti-MFF antibodies, respectively, followed by immunoblotting with anti-MFF or anti-CPT1A antibodies. **c** ATP concentration was assessed in SKOV-3 cells with control, CPT1A knockdown, and CPT1A knockdown in conjunction with CPT1A-H473A (H473A) overexpression, n=3 for each group. **d** CPT1A and MFF were examined by Western blotting in cells of (**c**). Beat-tubulin was used as a loading control. **e** The oxygen consumption rate (OCR) assay was performed in in SKOV-3 cell with control, CPT1A knockdown, or CPT1A knockdown in conjunction with CPT1A-H473A exogenous overexpression, n=3 for each group. **f, g** The maximal respiratory capacity (**f**) and reserved capacity (**g**) were quantified, n=3 for each group. **h** The EdU proliferation assay was performed in SKOV-3 cells with control, CPT1A knockdown, or CPT1A knockdown in conjunction with CPT1A-H473A exogenous overexpression. Representative image (left) and the ratio of EdU positive cells (n=3 for each group, right) are shown. Scale bars=25 μm. Data represent the mean ± SD; *p < 0.05, **p < 0.01 and ***p < 0.001, *n.s.* indicates no significant difference compared with the CPT1A-sh groups.

Supplementary Fig. 6

Fig. 1e

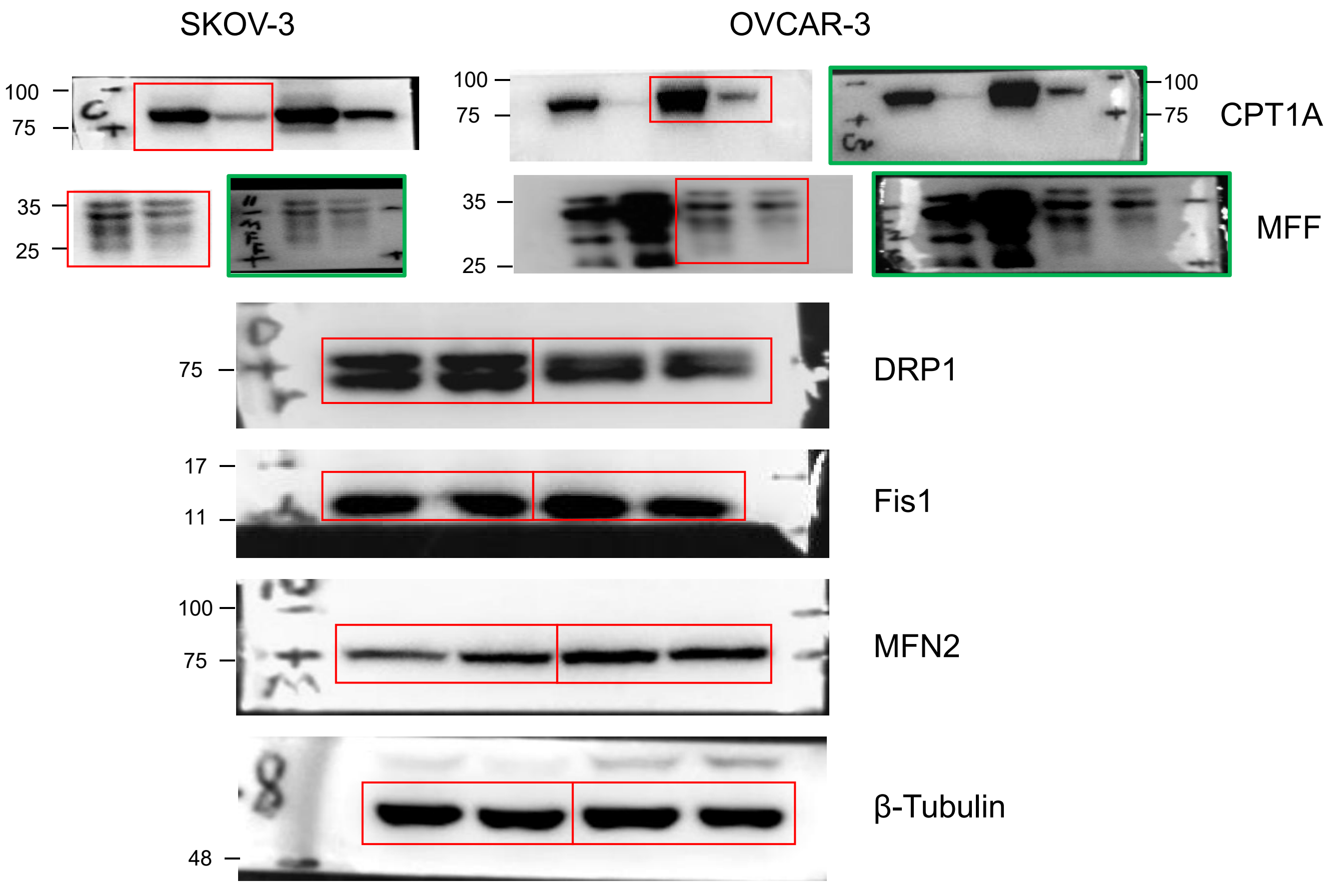

Fig. 1f

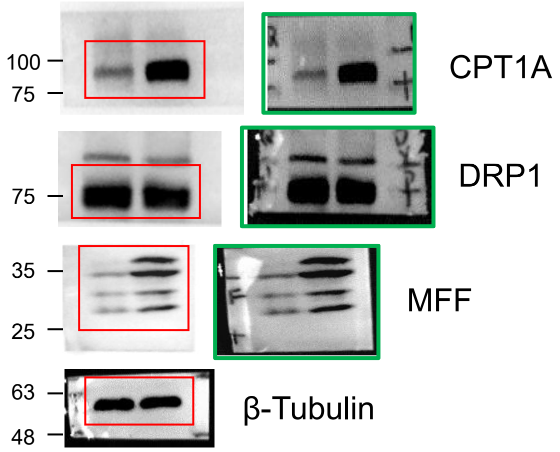

Fig. 2a

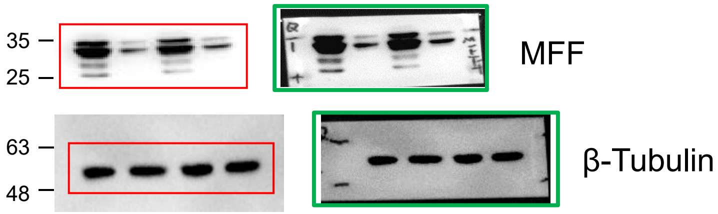

Fig. 3a

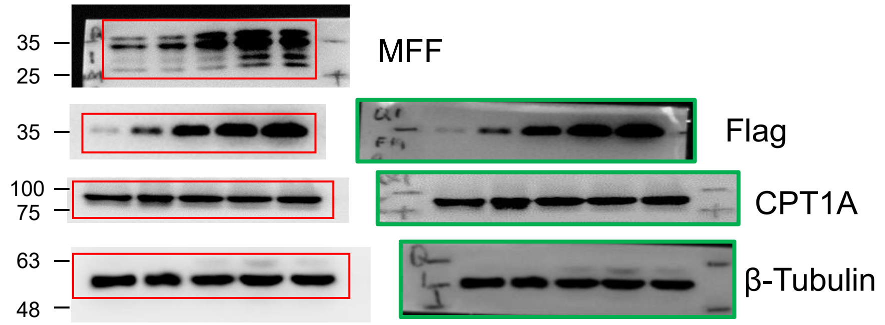

Fig. 3b

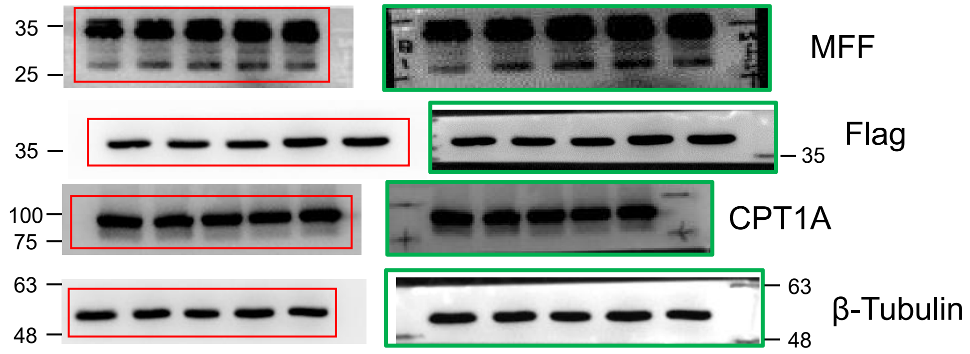

Fig. 3c

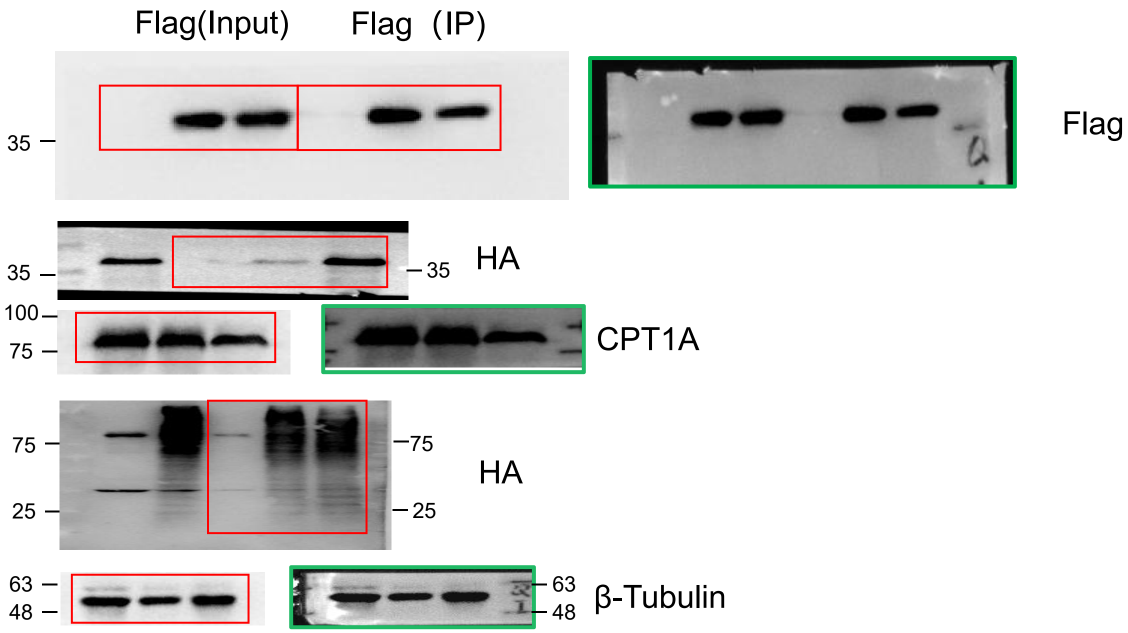

Fig. 3d

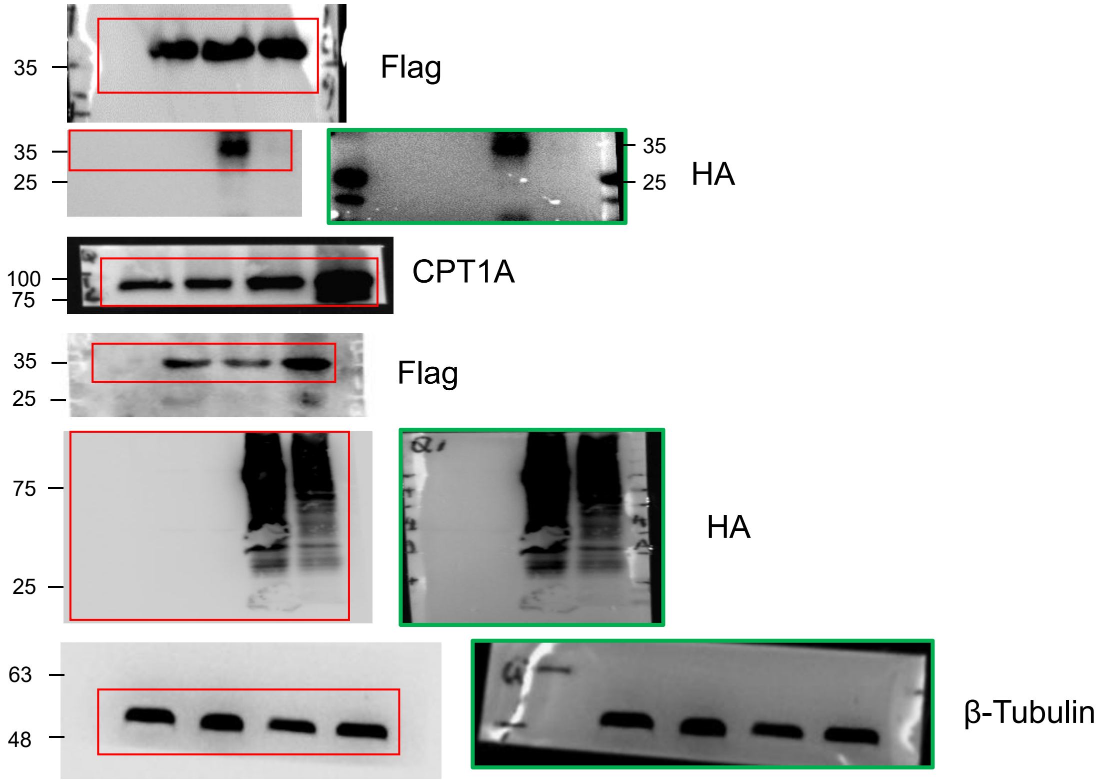

35
Flag
35
25
35
25
HA
100
75
CPT1A
35
25
Flag
75
25
HA
63
48
β-Tubulin

Fig. 3g

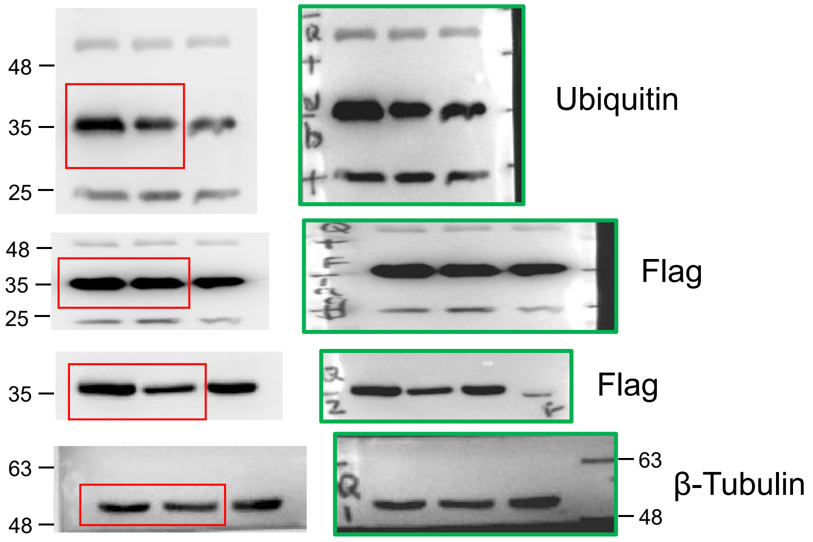

48
35
25
Ubiquitin
48
35
25
Flag
35
Flag
63
48
63
48
β-Tubulin

Fig. 3h

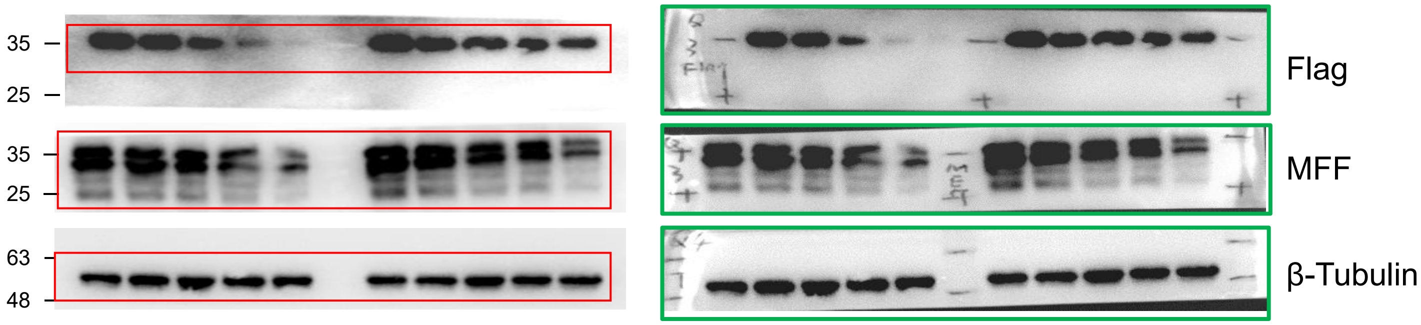

Fig. 4b

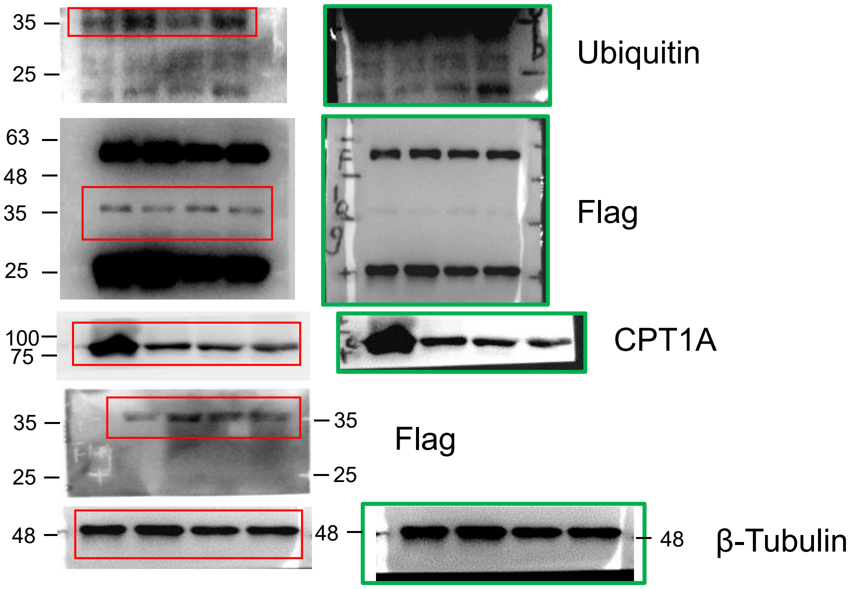

Fig. 4c

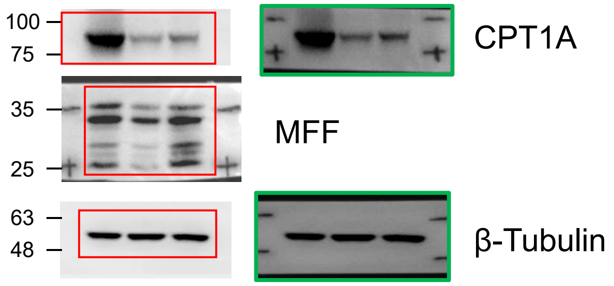

Fig. 4d

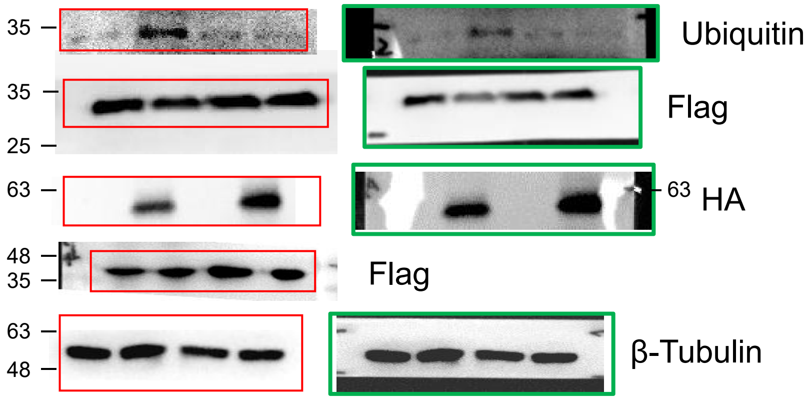

Fig. 4e

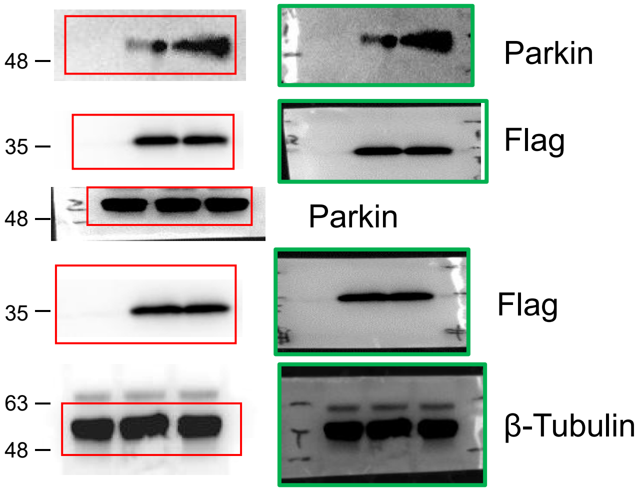

Fig. 4f

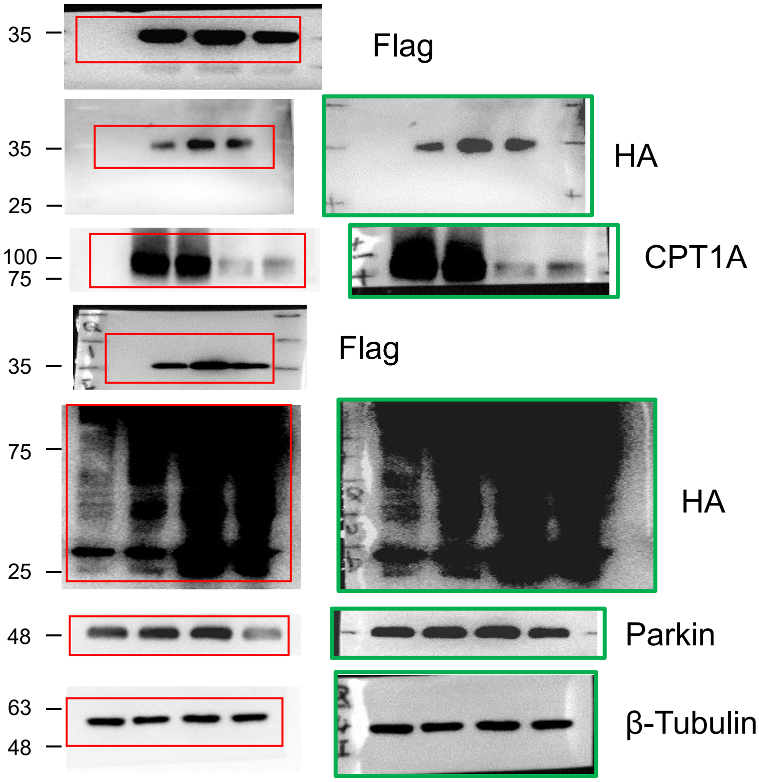

Fig. 5a

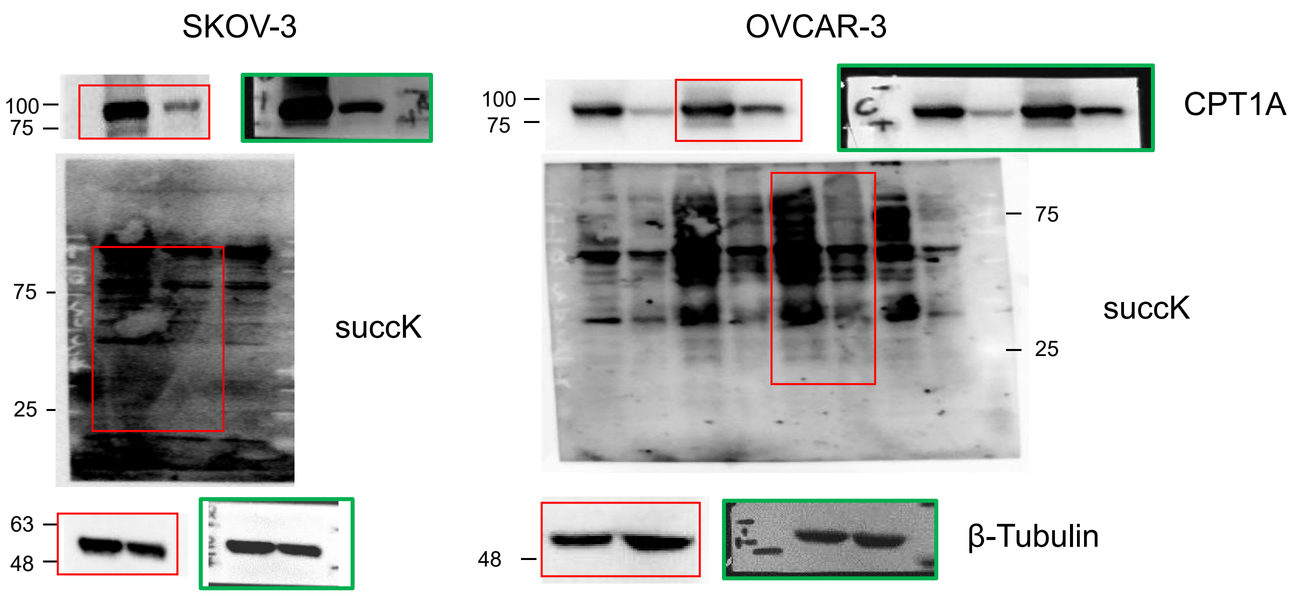
SKOV-3
OVCAR-3
100
75
CPT1A
75
25
succK
75
25
succK
63
48
48
β-Tubulin

Fig. 5b

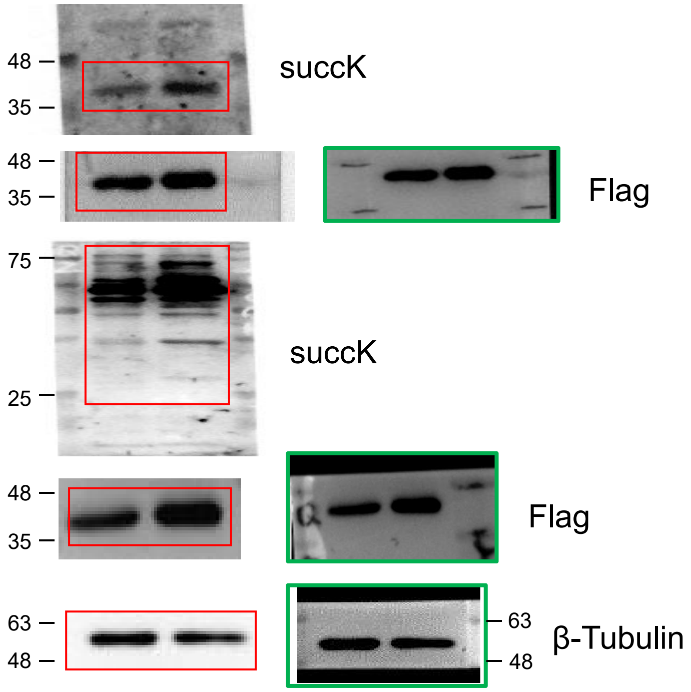
48
35
succK
48
35
Flag
75
25
succK
48
35
Flag
63
48
63
48
β-Tubulin

Fig. 5c

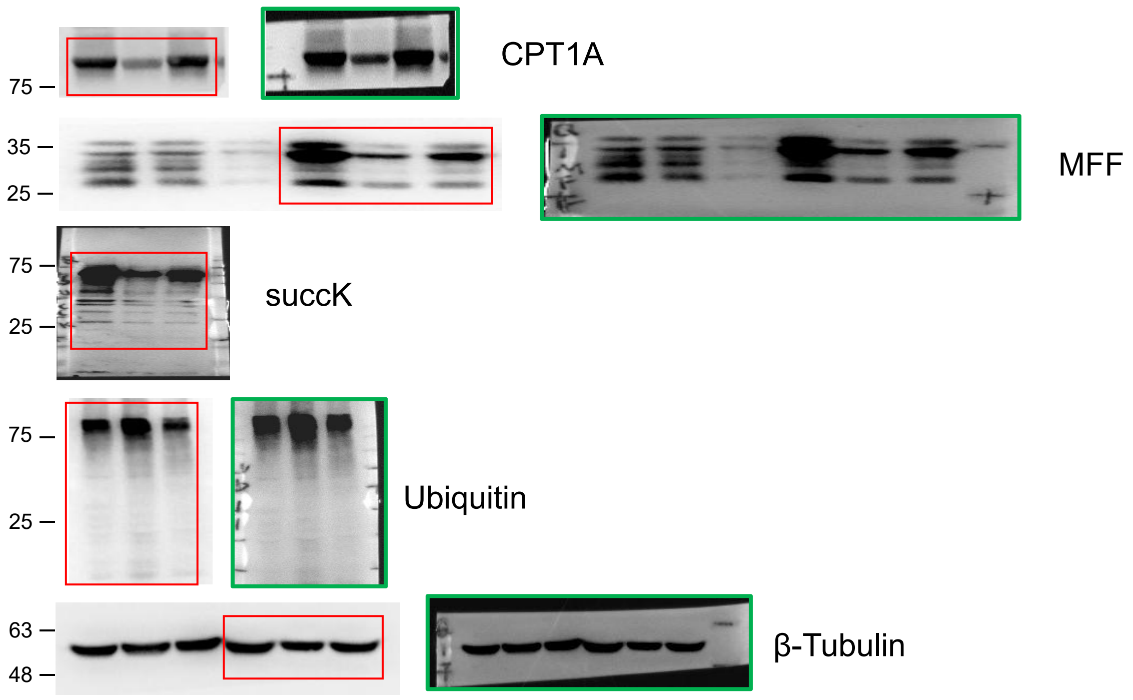

75
35
25
75
25
75
25
63
48
CPT1A
MFF
succK
Ubiquitin
β-Tubulin

Fig. 5d

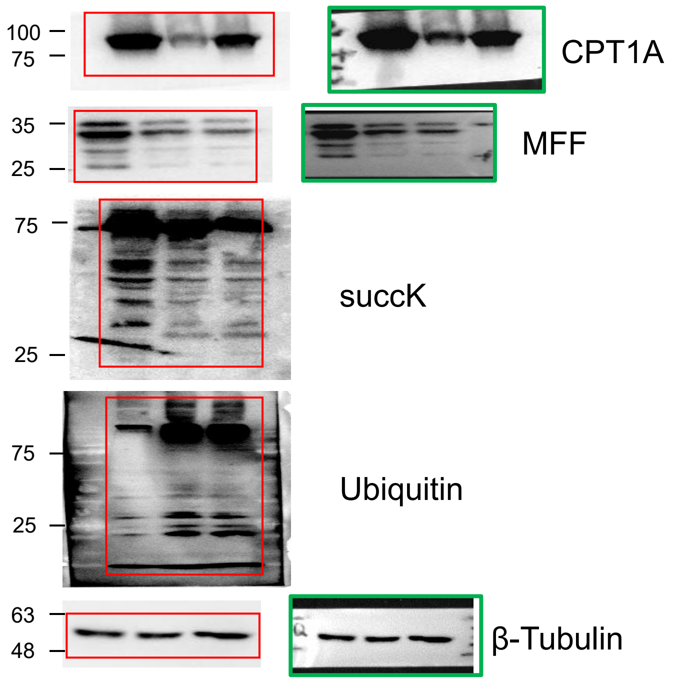

100
75
35
25
75
25
75
25
63
48
CPT1A
MFF
succK
Ubiquitin
β-Tubulin

Fig. 5g

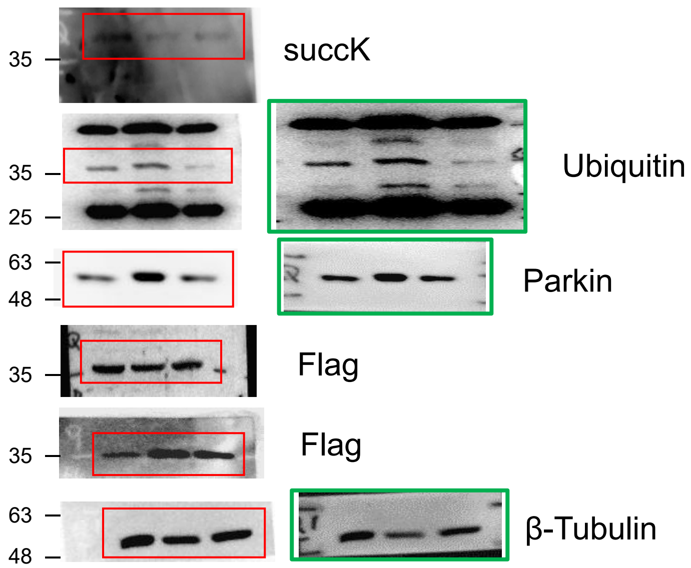

Fig. 5h

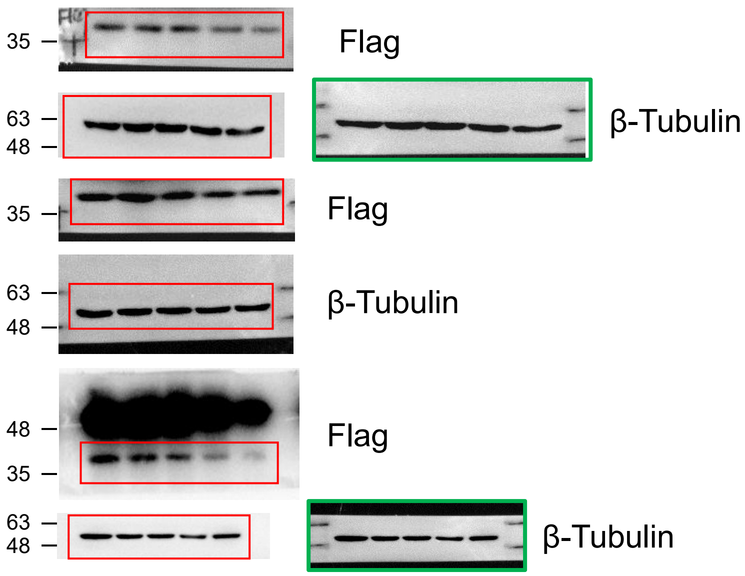

Fig. 6a

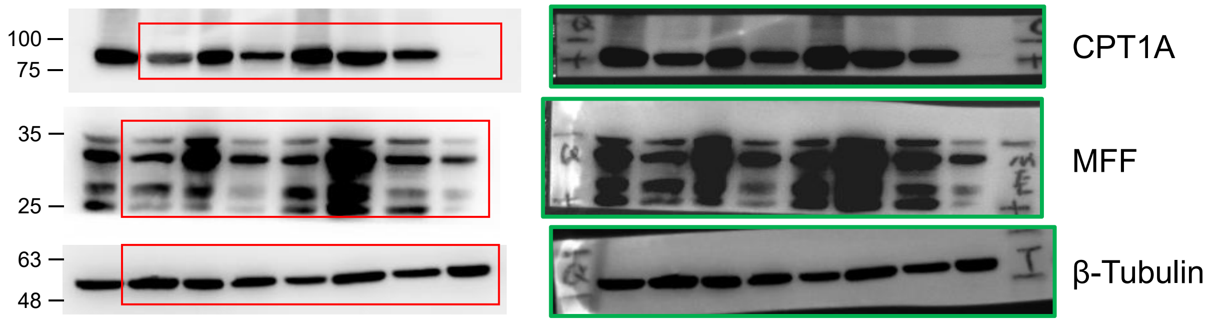

Fig. S1a

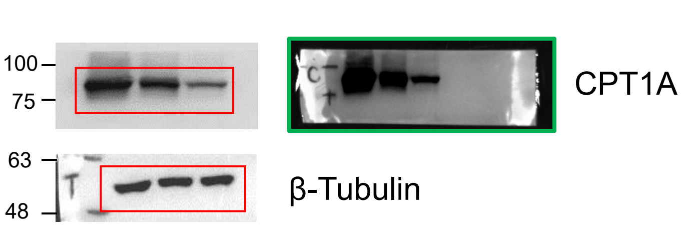

Fig. S1h

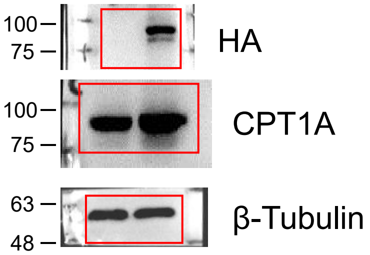

Fig. S1j

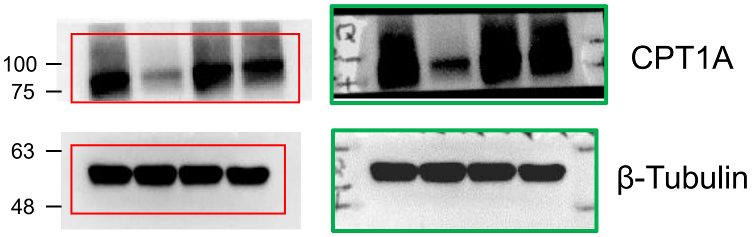

Fig. S1k

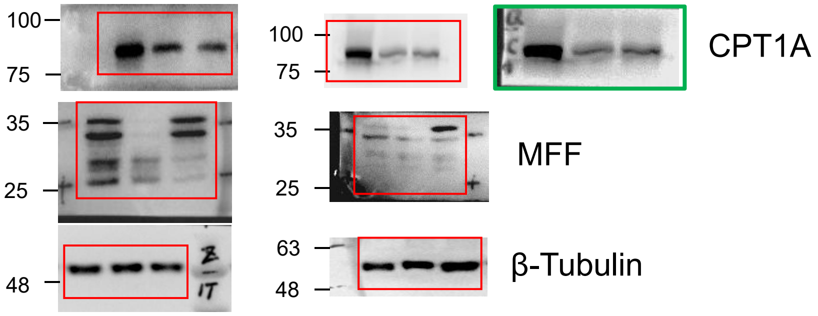

Fig. S1l

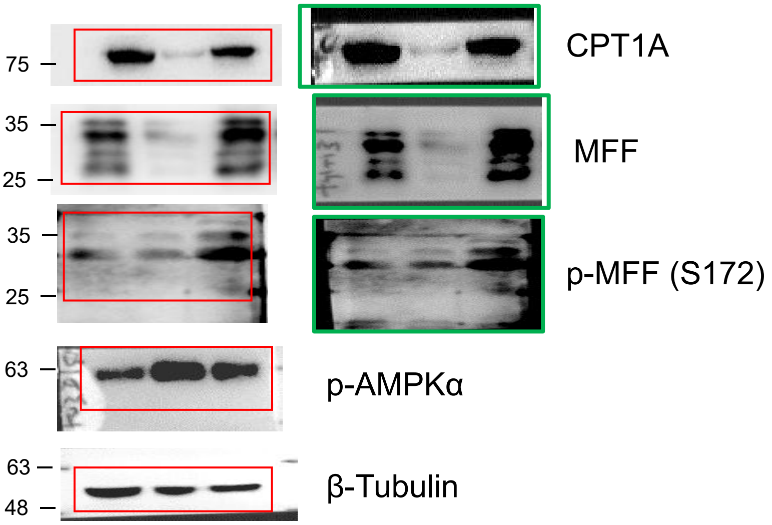

Fig. S2b

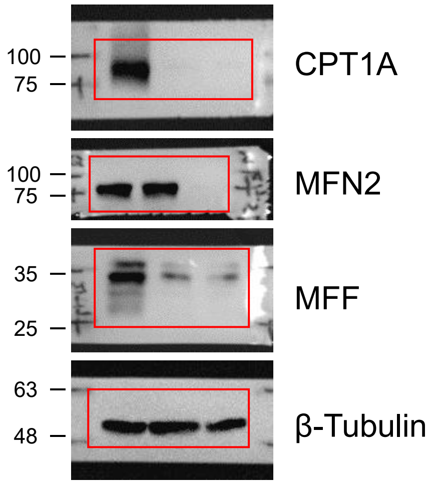

Fig. S2e

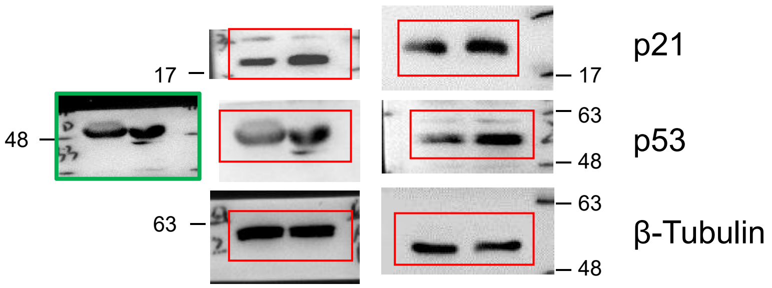

Fig. S2f

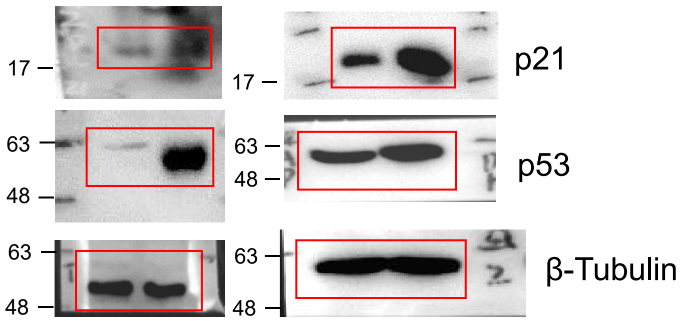

Fig. S2h

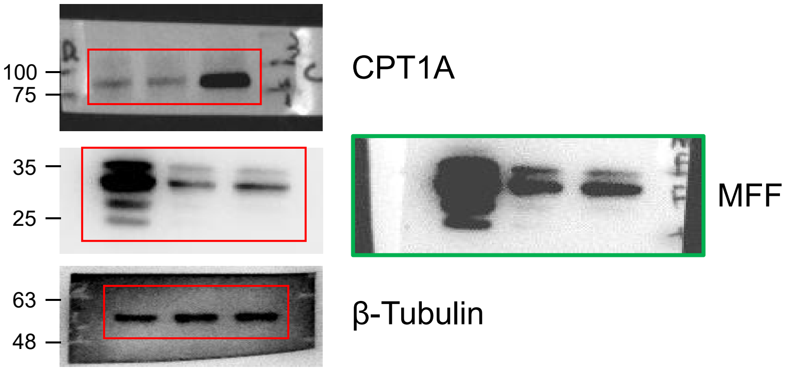

Fig. S3a

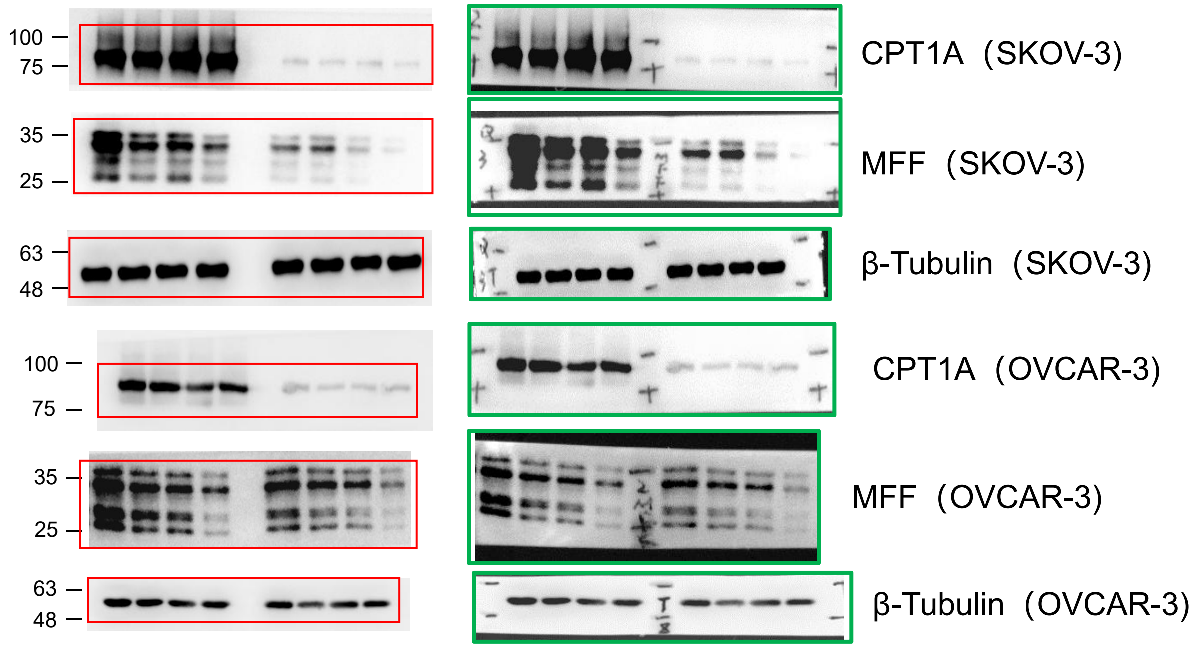

Fig. S3b

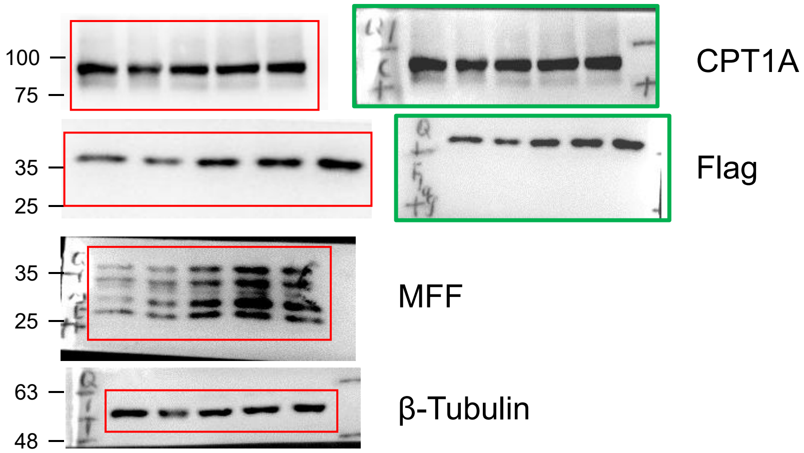

Fig. S3c

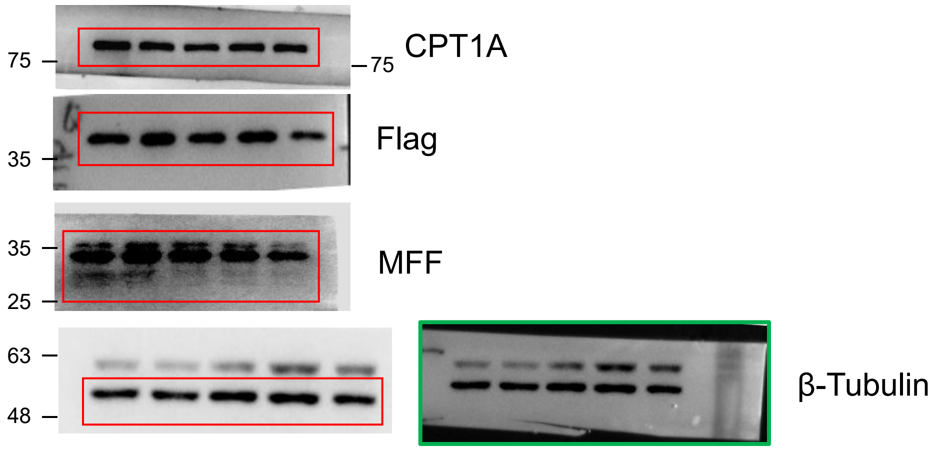

Fig. S3d

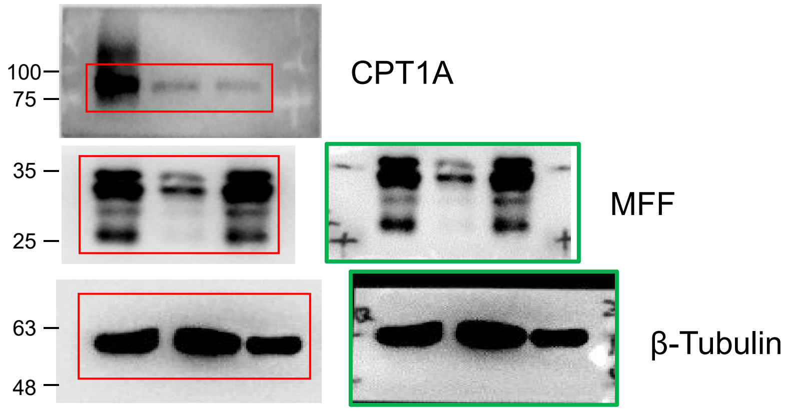

Fig. S5a

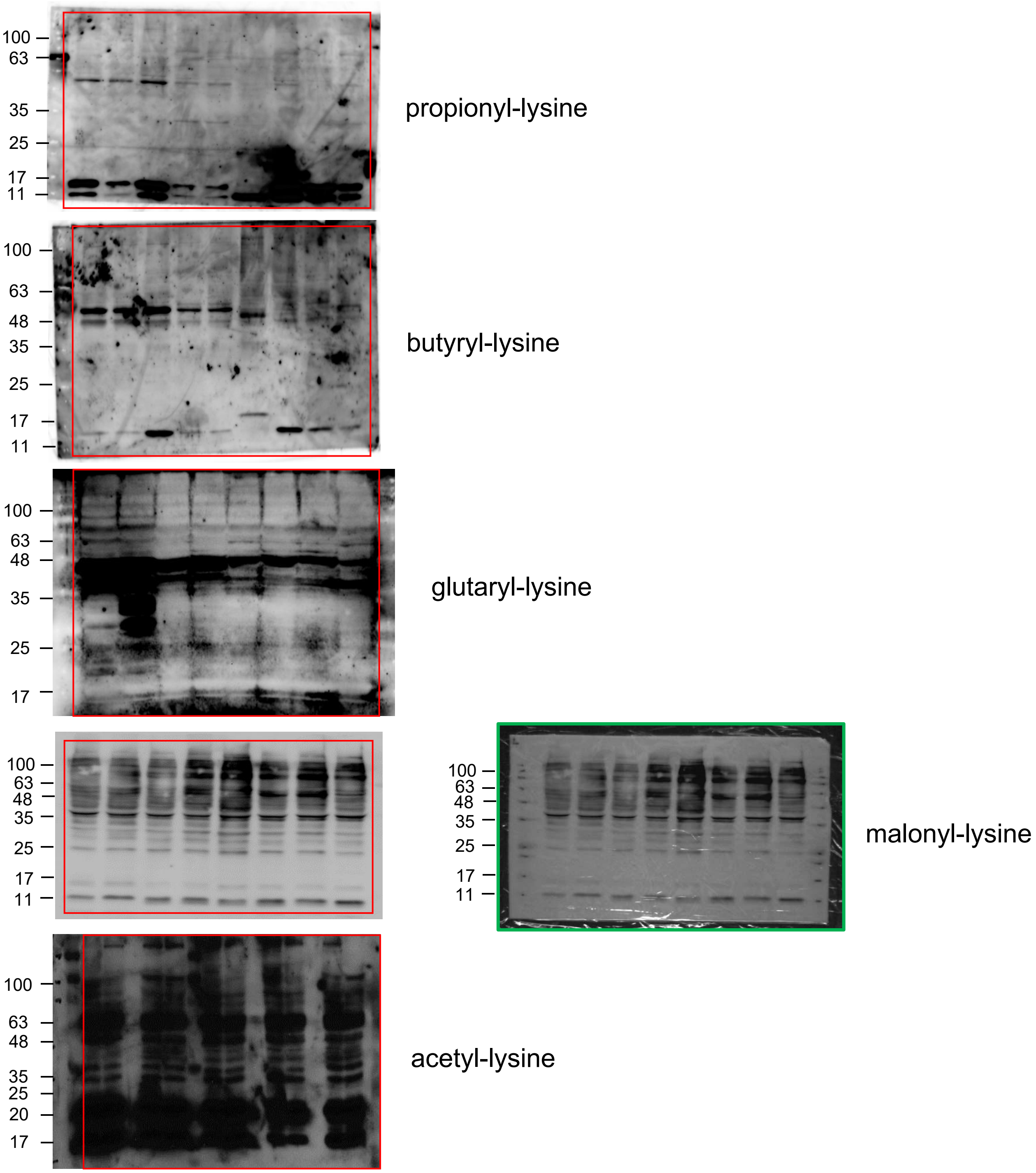

Fig. S5b

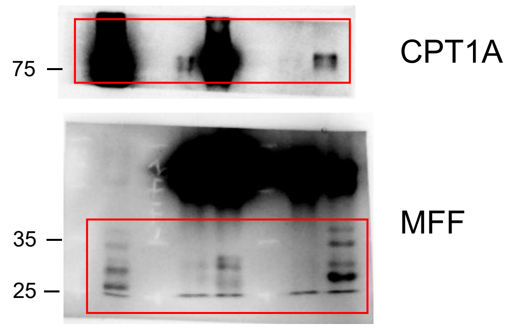

Fig. S5d

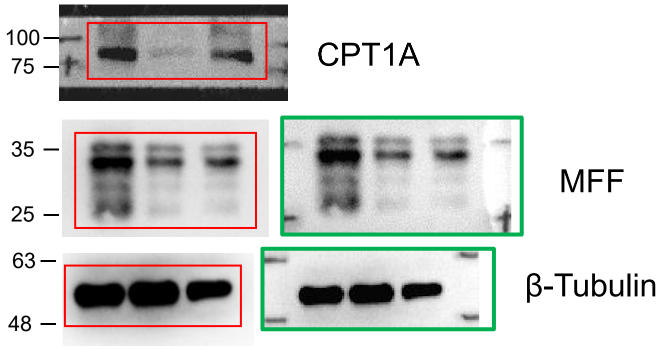

Supplementary Fig. 6. Uncropped/unedited images of blots.

Uncropped images of blots in the Figures and Supplementary Figures. The blots boxed in green are the corresponding ones with molecular weight ladders with different exposure time.

Supplementary Table 1: Antibody information

Antibodies used in this study					
Antibody	Species	Source	Cat no	Used in	Dilution
CPT1A	mouse	Abcam	128568	Western Blot	1:1000
				Immunohistochemistry	1:200
Drp1	rabbit	CST	5391	Western Blot	1:1000
				Immunofluorescence	1:200
Flag	mouse	Sigma	F1804	Western Blot	1:1000
Fis1	rabbit	Proteintech	10956-1-AP	Western Blot	1:1000
HA	rabbit	CST	3724	Western Blot	1:1000
Ki-67	mouse	Servicebio	111141	Immunohistochemistry	1:500
MFF	rabbit	CST	84580	Western Blot	1:1000
MFF	rabbit	Proteintech	17090-1-AP	Immunohistochemistry	1:800
MFN2	rabbit	CST	9482	Western Blot	1:1000
Parkin	rabbit	CST	2132	Western Blot	1:1000
Succinyllysine	rabbit	PTM BIO	PTM-401	Western Blot	1:1000
β-Tubulin	rabbit	Proteintech	11224-1-AP	Western Blot	1:4000
Ubiquitin	mouse	CST	3933	Western Blot	1:1000
p21	rabbit	CST	2947	Western Blot	1:1000
				Immunohistochemistry	1：50
p53	mouse	Proteintech	60283-2-Ig	Western Blot	1:1000
Propionyl-Lysine	rabbit	PTM BIO	PTM-201	Western Blot	1:1000
Malonyl-Lysine	rabbit	PTM BIO	PTM-901	Western Blot	1:1000
Acetylated-Lysine	rabbit	CST	9441S	Western Blot	1:1000
Glutaryl-Lysine	rabbit	PTM BIO	PTM-1151	Western Blot	1:1000
Butyryl-Lysine	rabbit	PTM BIO	PTM-301	Western Blot	1:1000
