# Supplementary material for: Carnitine palmitoyltransferase 1A promotes mitochondrial fission by enhancing MFF succinylation in ovarian cancer
Source: Commun Biol. 2023 Jun 8;6:618. doi: 10.1038/s42003-023-04993-x (PMC10250469; doi:10.1038/s42003-023-04993-x)
Supplement: Supplementary file 2 — Description of Additional Supplementary Files [file 42003_2023_4993_MOESM2_ESM.pdf]

Description of Additional Supplementary Files

File name: Supplementary Data 1

Description: The source data behind the graphs in the paper.

File name: Supplementary Data 2

Description: The ovarian cancer patient tissue microarrays details.
