# Supplementary material for: Carnitine palmitoyltransferase 1A promotes mitochondrial fission by enhancing MFF succinylation in ovarian cancer
Source: Commun Biol. 2023 Jun 8;6:618. doi: 10.1038/s42003-023-04993-x (PMC10250469; doi:10.1038/s42003-023-04993-x)
Supplement: Supplementary file 5 — Reporting Summary [file 42003_2023_4993_MOESM5_ESM.pdf]

nature portfolio

Corresponding author(s): Huanjie Shao

Last updated by author(s): May 30, 2023

Reporting Summary

Nature Portfolio wishes to improve the reproducibility of the work that we publish. This form provides structure for consistency and transparency in reporting. For further information on Nature Portfolio policies, see our Editorial Policies and the Editorial Policy Checklist.

Statistics

For all statistical analyses, confirm that the following items are present in the figure legend, table legend, main text, or Methods section.

n/a	Confirmed	
☐	☒	The exact sample size (*n*) for each experimental group/condition, given as a discrete number and unit of measurement
☐	☒	A statement on whether measurements were taken from distinct samples or whether the same sample was measured repeatedly
☐	☒	The statistical test(s) used AND whether they are one- or two-sided<br>*Only common tests should be described solely by name; describe more complex techniques in the Methods section.*
☒	☐	A description of all covariates tested
☒	☐	A description of any assumptions or corrections, such as tests of normality and adjustment for multiple comparisons
☐	☒	A full description of the statistical parameters including central tendency (e.g. means) or other basic estimates (e.g. regression coefficient) AND variation (e.g. standard deviation) or associated estimates of uncertainty (e.g. confidence intervals)
☐	☒	For null hypothesis testing, the test statistic (e.g. *F*, *t*, *r*) with confidence intervals, effect sizes, degrees of freedom and *P* value noted<br>*Give P values as exact values whenever suitable.*
☒	☐	For Bayesian analysis, information on the choice of priors and Markov chain Monte Carlo settings
☒	☐	For hierarchical and complex designs, identification of the appropriate level for tests and full reporting of outcomes
☐	☒	Estimates of effect sizes (e.g. Cohen's *d*, Pearson's *r*), indicating how they were calculated

Our web collection on statistics for biologists contains articles on many of the points above.

Software and code

Policy information about availability of computer code

Data collection	ImageJ (v.1.8.0.172) was used for data collection.
Data analysis	ImageJ (v.1.8.0.172) and Graphpad prism 8.0 were used for data analysis.

For manuscripts utilizing custom algorithms or software that are central to the research but not yet described in published literature, software must be made available to editors and reviewers. We strongly encourage code deposition in a community repository (e.g. GitHub). See the Nature Portfolio guidelines for submitting code & software for further information.

Data

Policy information about availability of data

All manuscripts must include a data availability statement. This statement should provide the following information, where applicable:

- Accession codes, unique identifiers, or web links for publicly available datasets
- A description of any restrictions on data availability
- For clinical datasets or third party data, please ensure that the statement adheres to our policy

All data generated or analyzed during this study are available from the corresponding author on reasonable request.

Human research participants

Policy information about studies involving human research participants and Sex and Gender in Research.

Reporting on sex and gender	Not applicable.
Population characteristics	Not applicable.
Recruitment	Not applicable.
Ethics oversight	Not applicable.

Note that full information on the approval of the study protocol must also be provided in the manuscript.

Field-specific reporting

Please select the one below that is the best fit for your research. If you are not sure, read the appropriate sections before making your selection.

☒ Life sciences ☐ Behavioural & social sciences ☐ Ecological, evolutionary & environmental sciences

For a reference copy of the document with all sections, see nature.com/documents/nr-reporting-summary-flat.pdf

Life sciences study design

All studies must disclose on these points even when the disclosure is negative.

Sample size	The sample size in this study was determined by reference to the literature on relevant experimental methods and the need for significance analysis.
Data exclusions	No data were excluded from the analyses.
Replication	All experiments were performed at least three times. We confirm that all results are reproducible.
Randomization	There are no samples/organisms/participants that require randomization in our study. However, we usually randomized images to eliminate subjective bias when analyzing those data.
Blinding	When we analyzed the effect of gene expression on mitochondrial dynamics, confocal microscope images were randomly numbered by one researcher and analyzed by another. Finally, the grouping is restored according to the initial number, and then the differences between different groups are statistically analyzed.

Reporting for specific materials, systems and methods

We require information from authors about some types of materials, experimental systems and methods used in many studies. Here, indicate whether each material, system or method listed is relevant to your study. If you are not sure if a list item applies to your research, read the appropriate section before selecting a response.

Materials & experimental systems

n/a	Involved in the study	
☐	☒	Antibodies
☐	☒	Eukaryotic cell lines
☒	☐	Palaeontology and archaeology
☐	☒	Animals and other organisms
☒	☐	Clinical data
☒	☐	Dual use research of concern

Methods

n/a	Involved in the study	
☒	☐	ChIP-seq
☒	☐	Flow cytometry
☒	☐	MRI-based neuroimaging

Antibodies

Antibodies used	Details of the antibodies used in this study are provided in the Supplementary Table S1.
Validation	All the antibodies were verified by the manufacturers and described in their website .

Eukaryotic cell lines

Policy information about cell lines and Sex and Gender in Research

Cell line source(s)	Cells were obtained from ATCC or Shanghai Qincheng Biotechnology Co., Ltd.
Authentication	Cells were confirmed by vendor via short tandem repeat profiling and were not reauthenticated by the authors.
Mycoplasma contamination	We confirm that the cells are not contaminated with mycoplasma.
Commonly misidentified lines (See ICLAC register)	We avoided using any of those commonly misidentified cell lines.

Animals and other research organisms

Policy information about studies involving animals; ARRIVE guidelines recommended for reporting animal research, and Sex and Gender in Research

Laboratory animals	BALB/c nude Mice (female), 5-6 weeks old.
Wild animals	Not applicable.
Reporting on sex	Ovarian cancer is a malignant tumor that develops in female reproductive system. So in this study, we chose female mice for tumor modeling.
Field-collected samples	Not applicable.
Ethics oversight	The experimental protocols were approved by the Institutional Animal Care and Use Committee of Shaanxi Normal University.

Note that full information on the approval of the study protocol must also be provided in the manuscript.
